# Checkpoint kinase 1 inhibition sensitises transformed cells to dihydroorotate dehydrogenase inhibition

**DOI:** 10.18632/oncotarget.19199

**Published:** 2017-07-12

**Authors:** Stéphanie Arnould, Geneviève Rodier, Gisèle Matar, Charles Vincent, Nelly Pirot, Yoann Delorme, Charlène Berthet, Yoan Buscail, Jean Yohan Noël, Simon Lachambre, Marta Jarlier, Florence Bernex, Hélène Delpech, Pierre Olivier Vidalain, Yves L. Janin, Charles Theillet, Claude Sardet

**Affiliations:** ^1^ Institut de Recherche en Cancérologie de Montpellier, Montpellier, France; ^2^ INSERM U1194, Montpellier, France; ^3^ Université de Montpellier, Montpellier, France; ^4^ Institut Régional du Cancer de Montpellier, Montpellier, France; ^5^ Réseau d'Histologie Expérimentale de Montpellier, BioCampus, UMS3426 CNRS-US009 INSERM-UM, Montpellier, France; ^6^ Montpellier RIO Imaging, BioCampus, UMS3426 CNRS-US009 INSERM-UM, Montpellier, France; ^7^ Laboratoire de Chimie et Biochimie Pharmacologiques et Toxicologiques, Equipe Chimie and Biologie, Modélisation et Immunologie pour la Thérapie, CNRS UMR 8601 CNRS-Université Paris Descartes, Paris, France; ^8^ Institut Pasteur, Unité de Chimie et Biocatalyse, CNRS UMR3523, Paris, France

**Keywords:** DHODH, Chk1, cytotoxicity, triple negative breast cancer, DNA damage

## Abstract

Reduction in nucleotide pools through the inhibition of mitochondrial enzyme dihydroorotate dehydrogenase (DHODH) has been demonstrated to effectively reduce cancer cell proliferation and tumour growth. The current study sought to investigate whether this antiproliferative effect could be enhanced by combining Chk1 kinase inhibition. The pharmacological activity of DHODH inhibitor teriflunomide was more selective towards transformed mouse embryonic fibroblasts than their primary or immortalised counterparts, and this effect was amplified when cells were subsequently exposed to PF477736 Chk1 inhibitor. Flow cytometry analyses revealed substantial accumulations of cells in S and G2/M phases, followed by increased cytotoxicity which was characterised by caspase 3-dependent induction of cell death. Associating PF477736 with teriflunomide also significantly sensitised SUM159 and HCC1937 human triple negative breast cancer cell lines to dihydroorotate dehydrogenase inhibition. The main characteristic of this effect was the sustained accumulation of teriflunomide-induced DNA damage as cells displayed increased phospho serine 139 H2AX (γH2AX) levels and concentration-dependent phosphorylation of Chk1 on serine 345 upon exposure to the combination as compared with either inhibitor alone. Importantly a similar significant increase in cell death was observed upon dual siRNA mediated depletion of Chk1 and DHODH in both murine and human cancer cell models. Altogether these results suggest that combining DHODH and Chk1 inhibitions may be a strategy worth considering as a potential alternative to conventional chemotherapies.

## INTRODUCTION

Dihydroorotate dehydrogenase (EC 1.3.5.2; DHODH) is the one mitochondrial enzyme that is located on the outer surface of the inner membrane and takes part in the fourth and rate-limiting step of *de novo* pyrimidine biosynthesis [1]. It converts dihydroorotic acid to orotic acid whilst reducing ubiquinone to ubiquinol which makes DHODH a link between pyrimidine synthesis and respiratory electron transport chain.

DHODH has emerged as a new therapeutic target in a wide spectrum of pathologies as *de novo* pyrimidine synthesis is extensively used in rapidly proliferating human or parasitic cells. Much effort has been devoted to designing new inhibitors in order to overcome widespread resistance to current antimalarial drugs [2–5] inasmuch as *Plasmodium* proliferation relies exclusively on this pathway [6]. Series of original compounds were also synthesised as part of a program aiming at identifying new antivirals [7–11] and a new compound is currently in clinical development for the treatment of fungal infection [12]. The immunosuppressant leflunomide has been prescribed for the treatment of inflammatory response associated with rheumatoid arthritis [13–16] and the immunomodulatory properties of its active metabolite teriflunomide (TFN; Supplementary Figure 1) led to its recent approval for the treatment of relapsing-remitting multiple sclerosis [17–19]. DHODH inhibition also effectively slowed down cancer cell and tumour growth of diverse tissue origins [20–25].

These inhibitors reduce dNTP pools available for DNA replication. Limiting precursors of DNA synthesis has been reported as a source of genetic instability [26–28] and reduced processivity of enzymes at replication forks or replication fork stalling [29, 30]. In order to prevent genetic instability, cells trigger a signalling pathway in which Chk1 effector kinase plays a crucial role through the activation of checkpoints in response to replication or genotoxic stress [31–33]. A wide array of chemotherapeutic drugs have been combined with Chk1 inhibitors in order to optimise treatment through the abrogation of checkpoints controlled by this kinase and allow accumulation of DNA damage that would jeopardize genome stability or induce cell death in a p53-compromised background [34]. Interestingly, our recent data [35] showed that upon knockout of E4F1 transcription factor transformed cells elicited major mitochondrial dysfunctions including a drastic reduction in levels of orotic acid and downstream pyrimidine intermediates. Furthermore E4F1 also controls the expression of *Chk1* gene, which results in a strong down-regulation of Chk1 protein expression and kinase activity in *E4F1KO* cells. We also observed that this combined down-regulation of mitochondrial and checkpoint activities strongly impacts on transformed cell survival, highlighting the potential interest of mimicking the deadly environment of *E4F1KO* cells by combining mitochondrial and checkpoint inhibitors.

This encouraged us to examine the association of Chk1 and *de novo* pyrimidine synthesis inhibitions as a new option to kill p53-deficient cancer cells.

## RESULTS

### Pharmacological activity of DHODH inhibitors in transformed mouse embryonic fibroblasts

**Figure 1:**
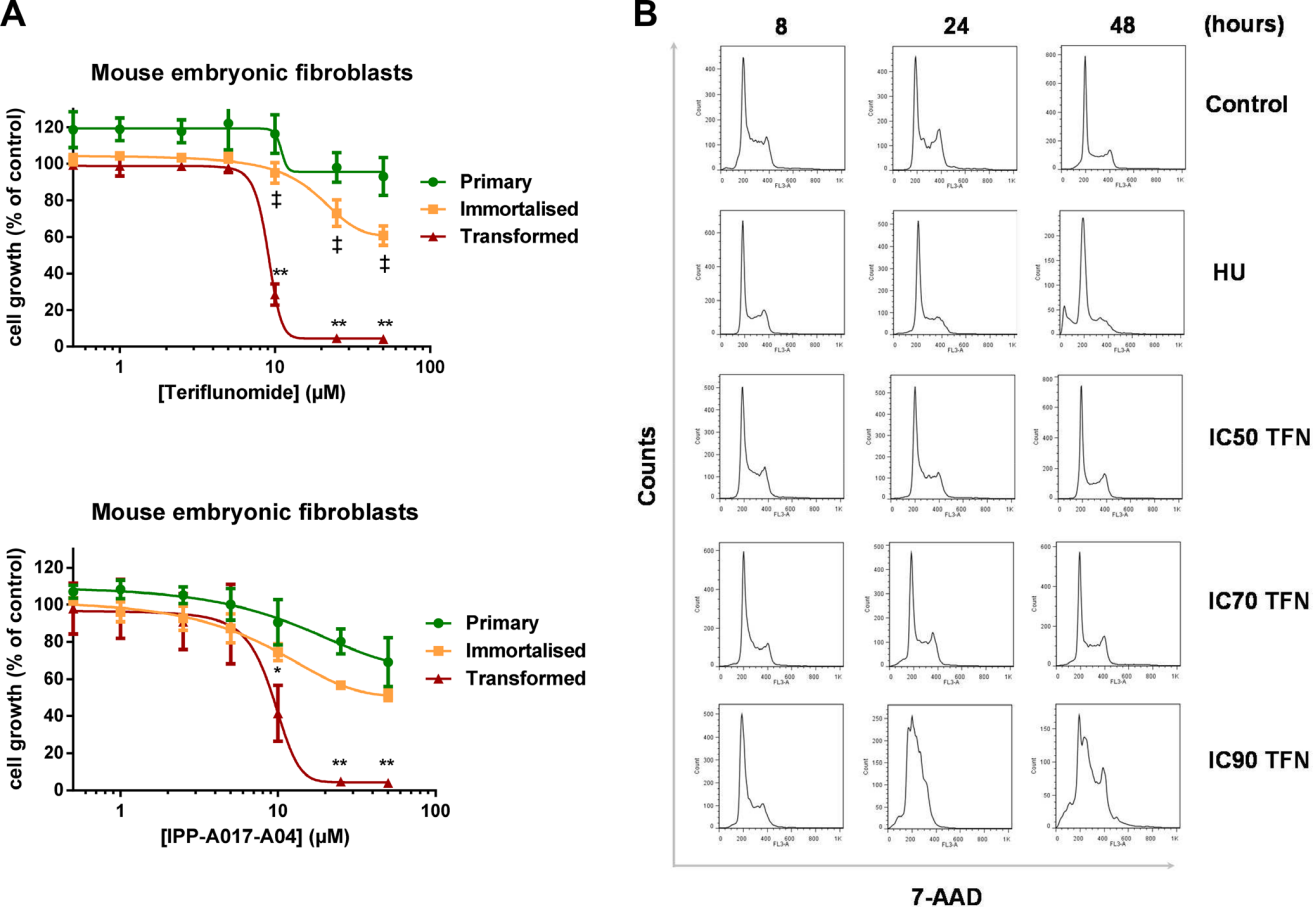
Selective pharmacological activity of teriflunomide in transformed mouse embryonic fibroblasts (**A**) Primary, immortalised and transformed MEFs were exposed to increasing concentrations of TFN or IPP-A017-A04 for 24 hours and grown in drug-free medium for three doubling times. Mean ± SD, *n* = 3 independent experiments. (**B**) Representative flow cytometry analysis of cell cycle distribution in populations of transformed MEFs collected at 8, 24 and 48 hours after the beginning of the exposure to ranging concentrations of TFN (IC50 = 8 μM ; IC70 = 10 μM ; IC90 = 25 μM) or 5 mM hydroxyurea (HU). *p < 0.05, **p < 0.01 when transformed mouse embryonic fibroblasts were compared with primary MEFs and ‡:*p* < 0.05 when transformed MEFs were compared with immortalised MEFs (as determined by two-tailed unpaired *t*-test).

The antiproliferative effect of DHODH inhibitor teriflunomide (TFN) was determined in primary, p53^KO^ and p53^KO^ mouse embryonic fibroblasts transformed by HaRas^V12^ derived from the same embryo [35] (Figure 1A). While a 24-hour exposure to TFN had a limited effect on primary and immortalised cells, it strongly reduced proliferation (monitored three doubling times after the end of this exposure) of transformed cells in a concentration-dependent manner (*p* < 0.01). This differential effect was also observed when these cell populations were exposed to another DHODH inhibitor, IPP-A017-A04 (Supplementary Figure 1; [10]), and the antiproliferative effect of both compounds was partly reversed by concomitant exposure to 50 μg/ml uridine (Supplementary Figure 2). To characterise this antiproliferative effect transformed MEFs were then exposed to TFN and cell cycle distribution was assessed for up to 48 hours (Figure 1B). Exposure to a high TFN concentration (IC90) induced a significant S phase accumulation at 24 hours followed with the appearance of a sub-G1 population at 48 hours. At lower concentrations such as IC50 and IC70 these effects were barely detectable at these time points.

### Pharmacological activity of Chk1 inhibitor PF477736 in combination with DHODH inhibition in primary, immortalised and transformed mouse embryonic fibroblasts

**Figure 2:**
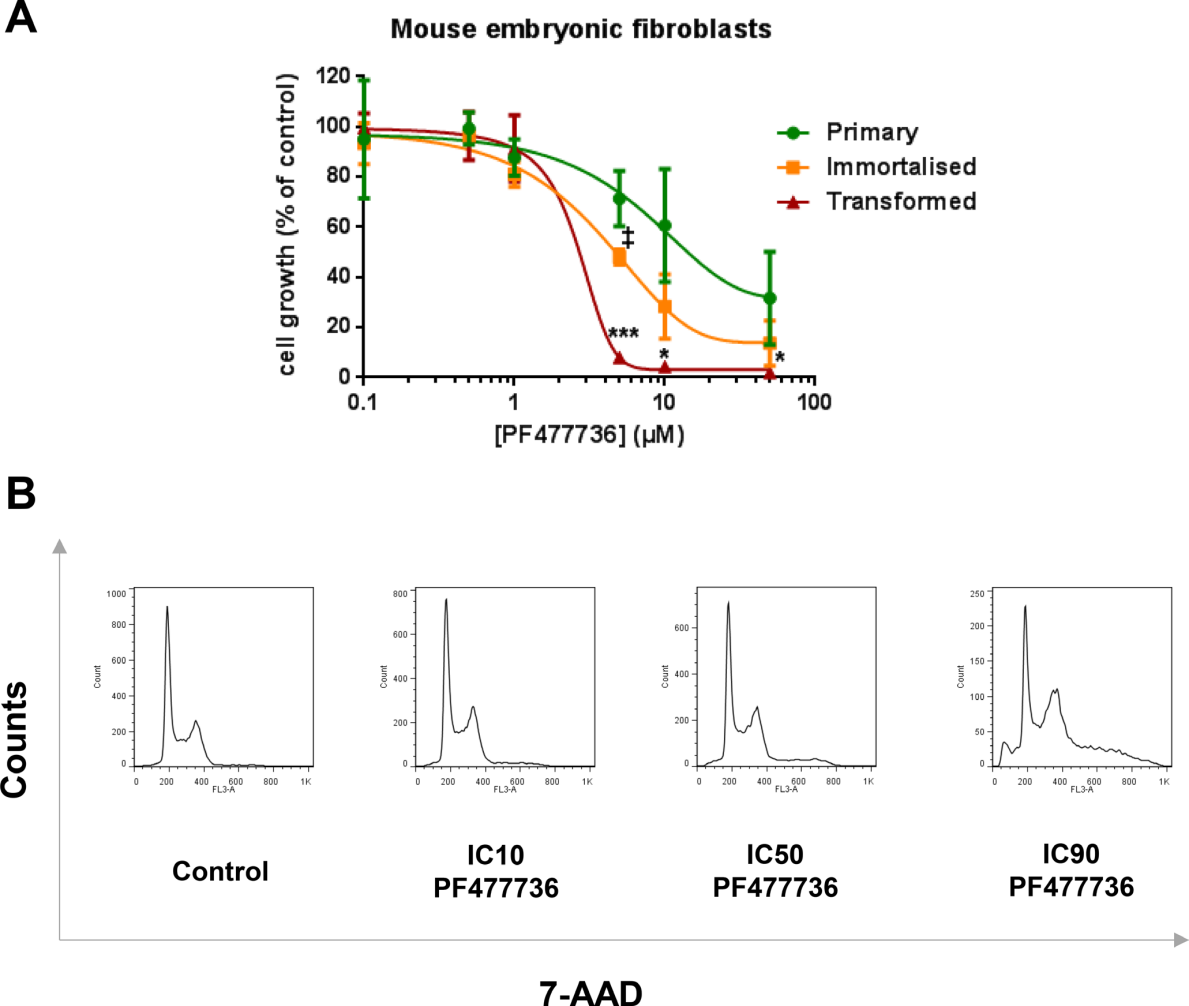
Selective pharmacological activity of PF477736 in transformed mouse embryonic fibroblasts (**A**) Primary, immortalised and transformed MEFs were exposed to increasing concentrations of PF477736 for 24 hours and grown in drug-free medium for three doubling times. Mean ± SD, *n* = 3 independent experiments. (**B**) Transformed MEFs were exposed to ranging concentrations of PF477736 (IC10 = 0.7 μM ; IC50 = 1.45 μM ; IC90 = 10 μM) for 24 hours and grown in drug-free medium. Representative flow cytometry analysis of cell cycle distribution performed 48 hours after the beginning of the exposure. *p < 0.05, ***p < 0.001 when transformed mouse embryonic fibroblasts were compared with primary MEFs and ‡:*p* < 0.05 when transformed MEFs were compared with immortalised MEFs (as determined by two-tailed unpaired *t*-test).

**Figure 3:**
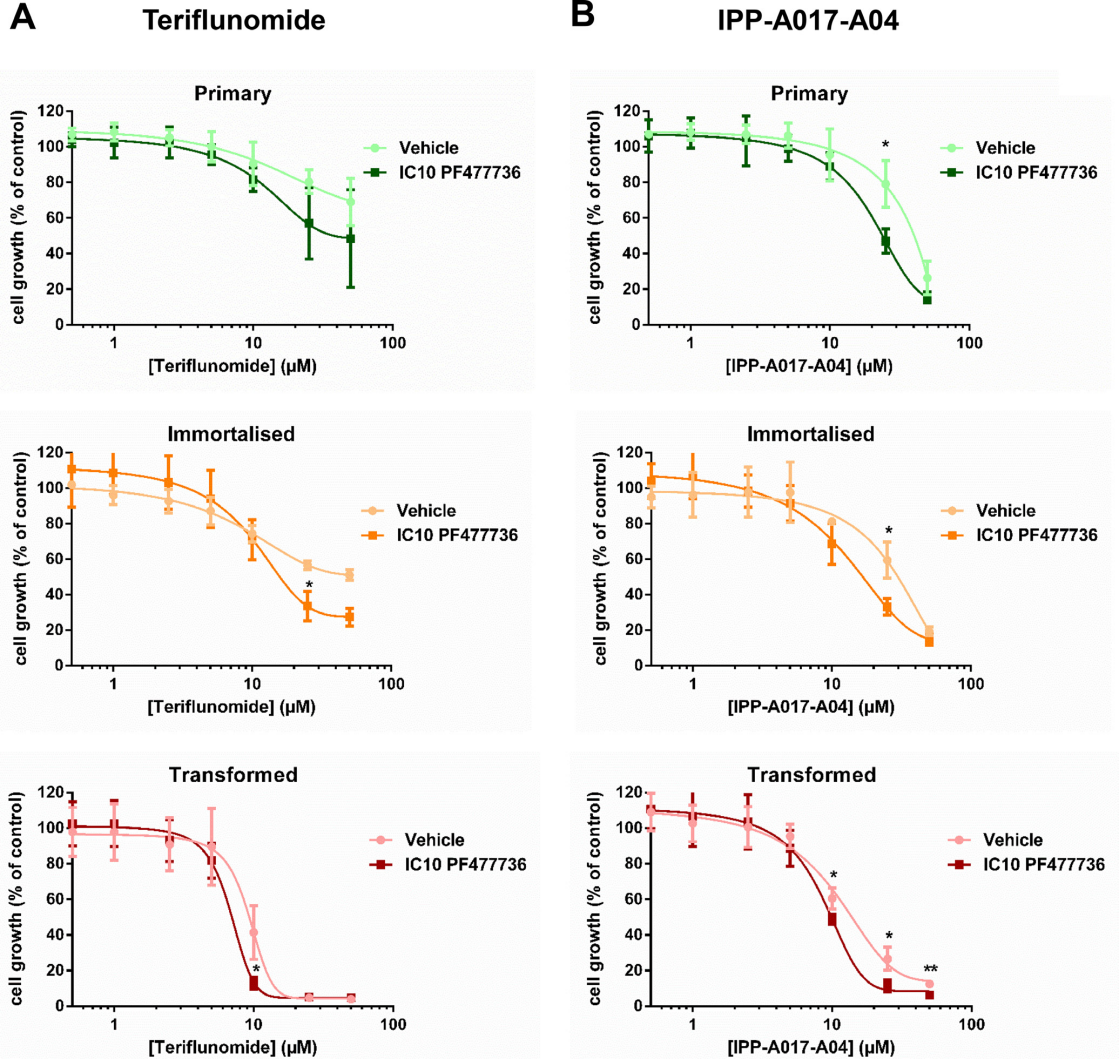
The combination of teriflunomide and PF477736 results in increased antiproliferative effect in transformed mouse embryonic fibroblasts Primary, immortalised or transformed mouse embryonic fibroblasts were exposed for 24 hours to increasing concentrations of teriflunomide ± transformed MEF IC10 PF477736 (0.7 μM) (which was added 30 minutes after the beginning of exposure to TFN) (**A**) or IPP-A017-A04 ± transformed MEF IC10 PF477736 (**B**), and grown in drug-free medium for three doubling times. Mean ± SD, *n* = 3 independent experiments. *p < 0.05, **p < 0.01 as determined by two-tailed unpaired *t*-test.

Primary, immortalised and transformed MEFs were also exposed to increasing concentrations of Chk1 inhibitor PF477736 (Supplementary Figure 1). Consistent with previous observations in human cancer cells [34], the antiproliferative effect of this compound was also more prominent in transformed MEFs than in their immortalised or primary counterparts (Figure 2A). These transformed fibroblasts were then exposed to ranging (IC10, IC50 and IC90) concentrations of PF477736 and cell cycle distribution was assessed at the 48-hour time point (Figure 2B). Profiles were indicative of a concentration-dependent cell cycle accumulation in S and G2/M phases along with the appearance, at the highest PF477736 concentrations, of hyperploid and sub-G1 cells. Of note these effects were undetectable upon exposure to IC10 PF477736. The same PF477736 concentration was then combined with ranging TFN concentrations to assess potentiation (Figure 3A). A moderate but significant potentiation effect was detected in immortalised and transformed cells (*n* = 3 independent experiments) suggesting that as little as IC10 PF477736 sensitised these cell models to DHODH inhibitors. Accordingly a similar potentiation effect of IC10 PF477736 occurred when combined with IPP-A017-A04 (Figure 3B).

**Figure 4:**
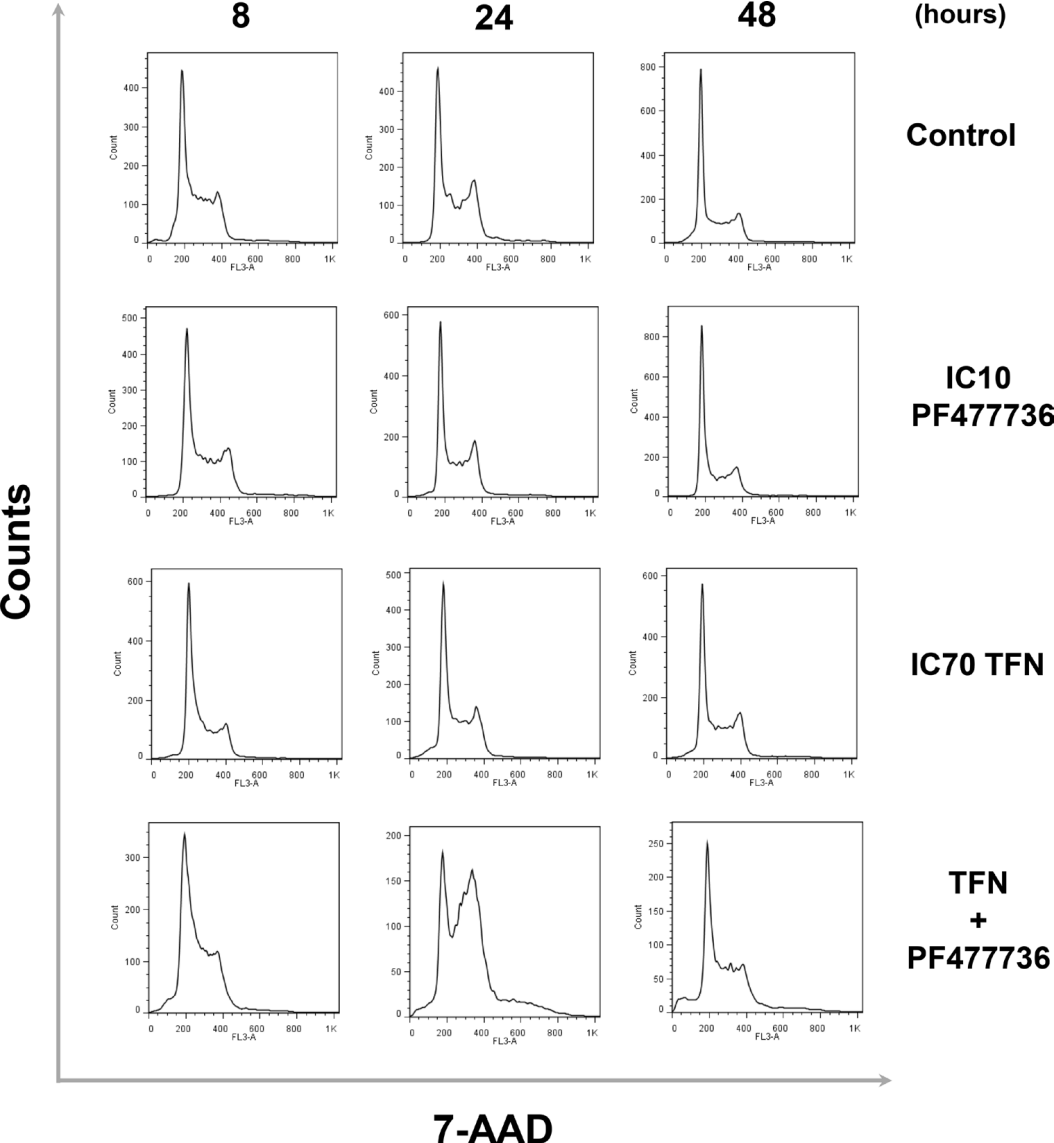
The combination of teriflunomide and PF477736 results in significant cell cycle perturbations in transformed mouse embryonic fibroblasts Representative flow cytometry analysis of cell cycle distribution in populations of transformed MEFs that were exposed for up to 24 hours to either IC70 TFN (10 μM) , IC10 PF477736 (0.7 μM) or the combination of these compounds at the same concentrations and collected at 8, 24 and 48 hours after the beginning of the time course.

In transformed cells this potentiation effect was significant (*p* = 0.0328) when IC10 PF477736 was combined with 10 μM TFN (IC70) (Figure 3A). Cell cycle distribution was therefore monitored at these optimal concentrations for up to 48 hours (Figure 4). Cells exposed to this combination already accumulated in S phase 8 hours after the beginning of the time course. At 24 hours they further accumulated in S as well as G2/M. At this time point a substantial hyperploid population was also detectable. Finally, at a later time point (48 h), sub-G1 cells were clearly detected upon exposure to this TFN + PF477736 combination. Importantly, neither cell cycle perturbation nor cell death was observed with either inhibitor alone. A similar trend was noticed in cells exposed to the IPP-A017-A04 + PF477736 combination (Supplementary Figure 3).

### DNA damage signalling in response to DHODH and Chk1 inhibitors in transformed mouse embryonic fibroblasts

Nucleotide depletion is a major cause of replication stress and DNA damage. Cells cope with these insults by triggering a protective signalling pathway that involves Chk1 kinase activation by phosphorylation. Thus the combination of antimetabolites such as gemcitabine and Chk1 inhibitors was reported to induce DNA double strand breaks [36]. We therefore hypothesized that cells exposed to DHODH and Chk1 inhibitors would accumulate DNA damage as monitored by the presence of γH2AX staining (H2AX phosphorylation on serine 139) and ATR-dependent phosphorylation of Chk1 on serine 345. Gamma H2AX staining and Chk1 phosphorylation were indeed reported as pharmacodynamic markers of chemopotentiation and Chk1 inhibition in combinations of genotoxic drugs and Chk1 inhibitors [37].

**Figure 5:**
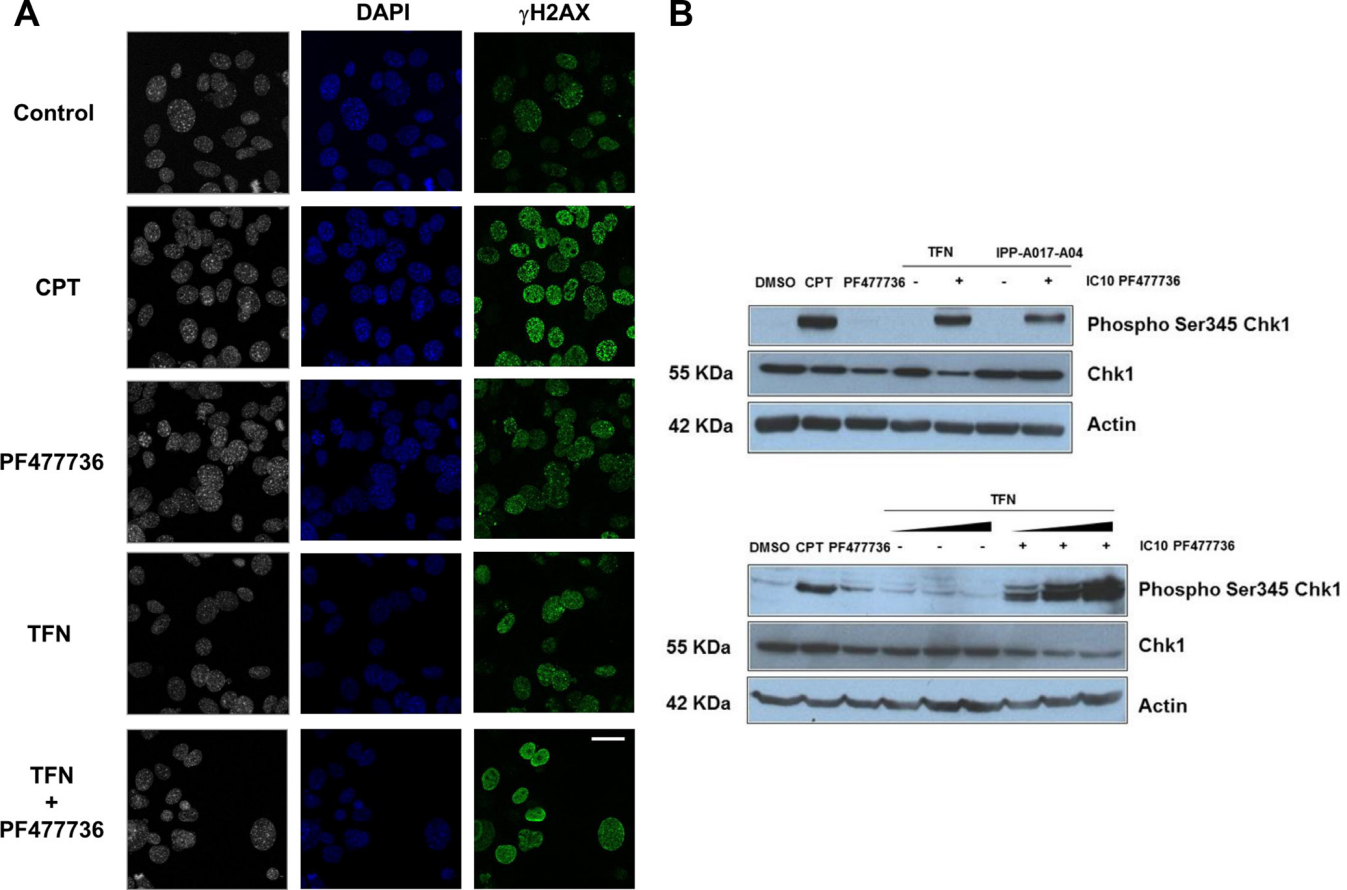
The combination of teriflunomide and PF477736 increases the amount of DNA damage in transformed MEFs (**A**) Immunofluorescence of DNA (blue) and γH2AX (H2AX phosphorylation on serine 139) (green) in transformed MEFs that were exposed to either IC70 TFN (10 μM), IC10 PF477736 (0.7 μM), the combination of these compounds or 0.1 μM positive control camptothecin (CPT). Scale bar, 20 μm. (**B**) Western blotting analysis of Chk1 phosphorylation on serine 345 in cell lysates prepared 8 hours after the beginning of the exposure. Upper panel: transformed MEFs were exposed to vehicle, 0.1 μM camptothecin, IC10 PF477736, IC70 TFN ± IC10 PF477736 and IC70 IPP-A017-A04 (22 μM) ± IC10 PF477736. Lower panel: cells were exposed to vehicle, 0.1 μM camptothecin, IC70 TFN, IC10 PF477736, or increasing (IC50 = 1.45 μM, IC70 = 10 μM and IC90 = 25 μM) concentrations of teriflunomide ± IC10 PF477736.

While the amount and intensity of γH2AX foci were limited in transformed cells exposed to IC70 TFN or IC10 PF477736 alone, cell exposure to the combination of these inhibitors resulted in a significant increase in γH2AX staining (Figure 5A), indicative of substantial DNA damage. Consistently the phosphorylation of Chk1 on serine 345 was also strongly stimulated in these TFN + PF477736-treated cells (Figure 5B) whereas it was undetectable in cells exposed to either inhibitor alone. Similar results were obtained when cells were exposed to a combination involving IPP-A017-A04 DHODH inhibitor. Interestingly, the intensity of Chk1 phosphorylation on serine 345 in the presence of PF477736 was dependent on TFN concentration (Figure 5B, lower panel). This suggested that Chk1 phosphorylation on serine 345, which is a hallmark of Chk1 activation, could be used as a quantitative marker of DHODH inhibition in this setting.

Of note DNA damage is also known to stimulate Chk2 phosphorylation [38] as exemplified here by exposure of transformed MEFs to camptothecin (CPT) used as a positive control (Supplementary Figure 4). Accordingly Chk2 phosphorylation was detected in lysates prepared from cells exposed to DHODH + Chk1 inhibitors.

### Cell death in transformed mouse embryonic fibroblasts exposed to the combination of DHODH and Chk1 inhibitors

**Figure 6:**
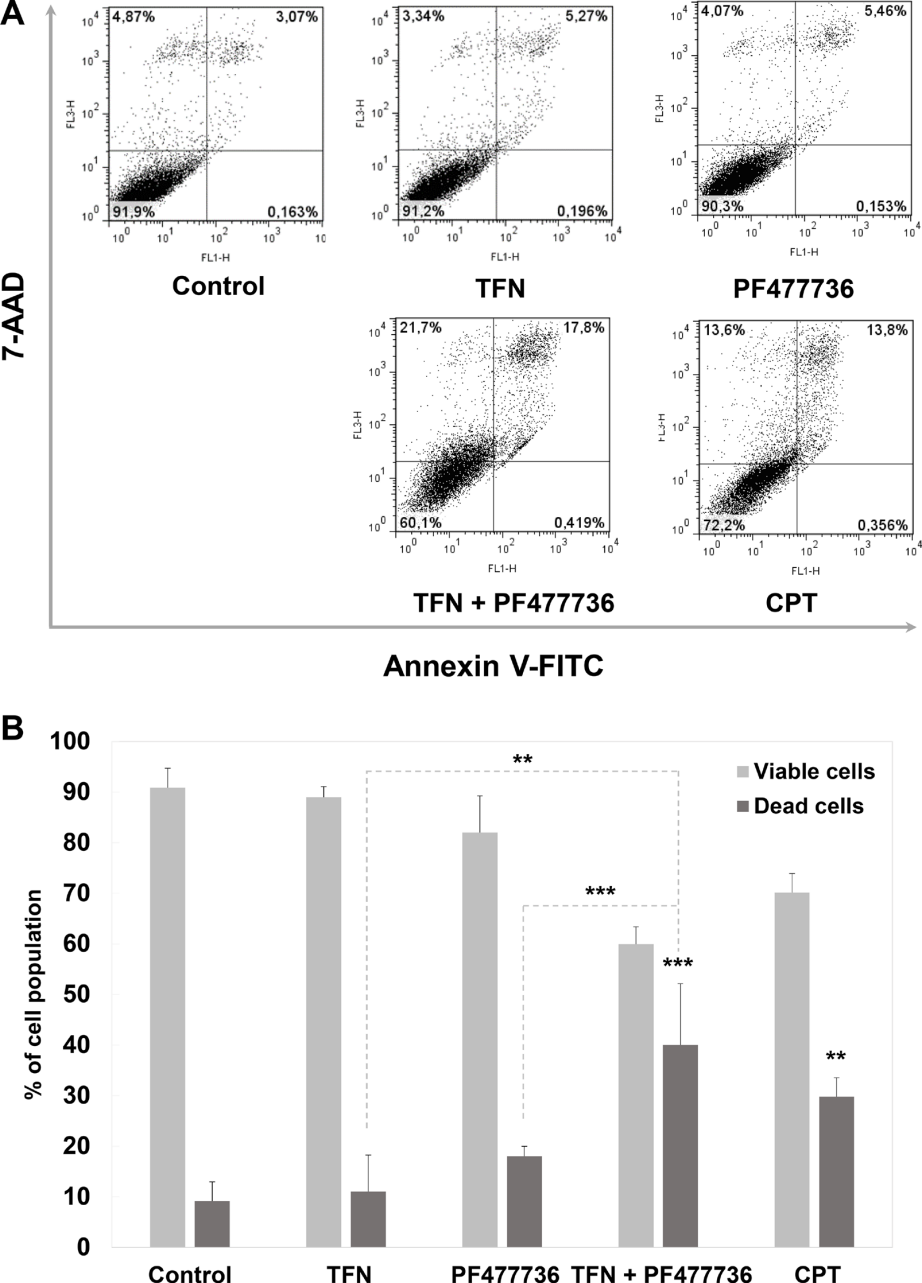
The combination of teriflunomide and PF477736 is cytotoxic in transformed mouse embryonic fibroblasts (**A**) Flow cytometry analysis for apoptosis / necrosis. Cells were exposed to vehicle, positive control camptothecin (0.1 μM), IC70 TFN (10 μM), IC10 PF477736 (0.7 μM) or their combination, collected and stained with annexin V/7AAD. Results are representative of three independent experiments. (**B**) Quantitation was performed with FlowJo software. Results are expressed as mean values ± SD of three independent experiments. **p < 0.01, ***p < 0.001 as determined by two-tailed unpaired *t*-test.

The induction of massive DNA damage suggested by γH2AX staining and Chk1/Chk2 phosphorylation prompted us to assess cell fate in response to these compounds and their combination. In order to assess cell death, annexin V/7-AAD staining was performed in cell populations that were collected 48 hours after the beginning of the time course. Dot plots (Figure 6A) showed a larger population of annexin V-positive/7-AAD-positive cells upon exposure to the combination as compared with matched controls or each individual inhibitor. These annexin V/7-AAD profiles were indicative of both apoptosis and necrosis. Upon quantification (Figure 6B), this increase in mortality was highly significant (*p* = 0.0002 as compared with TFN; *p* = 0.0089 as compared with PF477736 and *p* = 0.0245 as compared with camptothecin). Again a similar trend was noticed upon exposure to the IPP-A017-A04 + PF477736 combination as compared with single compounds or matched controls (Supplementary Figure 5).

In order to confirm that exposure to Chk1 and DHODH inhibitors results in similar phenotypic changes as the ones that occur upon loss of Chk1 and DHODH functions, transformed mouse embryonic fibroblasts were transfected with siRNA for Chk1 and DHODH and protein depletion was assessed using western blotting at 48 hours (Supplementary Figure 6A). Flow cytometry analysis showed a significant induction of cell death upon dual siRNA knockdown as compared with single depletions or control siRNA (Supplementary Figure 6B). These results suggested that the effects that were observed upon PF477736 and TFN exposure were representative of Chk1 and DHODH inhibitions respectively.

### Phenotypic effect of the combination of DHODH and Chk1 inhibitors in triple negative breast cancer cell lines

The cytotoxic effect that was observed in transformed mouse embryonic fibroblasts raised the question as to whether the combination of DHODH and Chk1 inhibitors would also be effective in a model of human cancer cells. Our interest was focused on triple negative breast cancer (TNBC) as this pathology is highly resistant to most conventional chemotherapies.

In order to ensure the selective effect of Chk1 inhibition in this strategy, SUM159 TNBC cells were exposed to increasing concentrations of TFN with or without a fixed concentration of PF477736. We compared the modulation of the antiproliferative effect of TFN by either 0.1 μM, 0.5 μM or 2.5 μM PF477736 (the latter corresponding to IC10 in this cell line, Supplementary Figure 7A). The chemopotentiation of TFN effect by PF477736 was dependent on the value of the fixed concentration of Chk1 inhibitor (Supplementary Figure 8A). The autophosphorylation of Chk1 on serine 296 that occurs upon DNA damage and is considered as a relevant biomarker for Chk1 kinase activity, was assessed by western blotting (Supplementary Figure 8B). SUM159 cells were exposed to either 0.1 μM, 0.5 μM or 2.5 μM PF477736 alone or in combination with IC70 TFN (or 40 μM gemcitabine as a positive control). Phospho-Ser296 Chk1 levels were reduced in a concentration-dependent manner, with the lowest level observed at 2.5 μM PF477736, which confirmed optimal kinase inhibition at that concentration. A concentration-dependent increase in phospho-Ser345 Chk1 levels was concurrently observed when Chk1 was inhibited (Supplementary Figure 8B). Thus the increase in the antiproliferative effect of TFN was dependent on PF477736 concentration up to the IC10, and the involvement of Chk1 inhibition in this phenomenon was confirmed by the concomitant decrease in the levels of Chk1 phosphorylation on serine 296 within the same concentration range.

**Figure 7:**
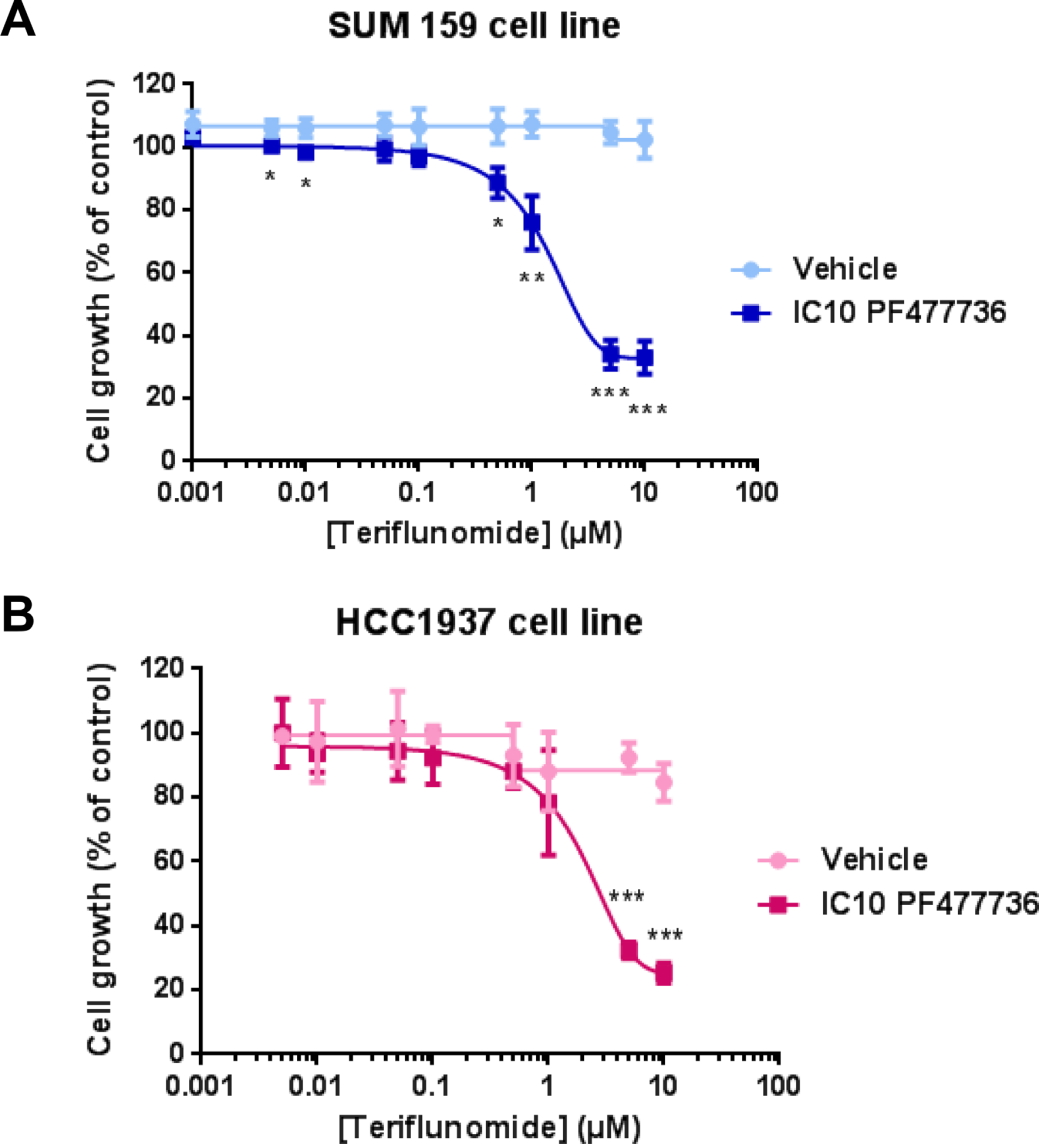
The combination of teriflunomide and PF477736 reduces proliferation of SUM159 and HCC1937 triple negative breast cancer cell lines (**A**) SUM159 and (**B**) HCC1937 cells were exposed for 24 hours to increasing concentrations of teriflunomide ± IC10 PF477736 (2.5 μM and 0.29 μM respectively, added 30 minutes after the beginning of exposure to TFN) and grown in drug-free medium for three doubling times. Mean ± SD, *n* = 3 independent experiments. *p < 0.05, **p < 0.01, ***p < 0.001 as determined by two-tailed unpaired *t*-test.

As our strategy was validated and the antiproliferative effect of PF477736 as a single agent was determined in a panel of four triple negative breast cancer cell lines including SUM159 (Supplementary Figure 7A), each cell model was exposed to increasing TFN concentrations with or without IC10 PF477736. While TFN alone had almost no effect in SUM159 cell line, its combination with IC10 Chk1 inhibitor resulted in a drastic decrease in cell growth (Figure 7A), and the same phenomenon was observed in HCC1937 cell line (Figure 7B). In contrast this combination had no significant effect in either BT549 (Supplementary Figure 7B) or HCC38 (Supplementary Figure 7C) cell lines which were however more sensitive to PF477736 than the aforementioned cell lines (Supplementary Figure 7A).

**Figure 8:**
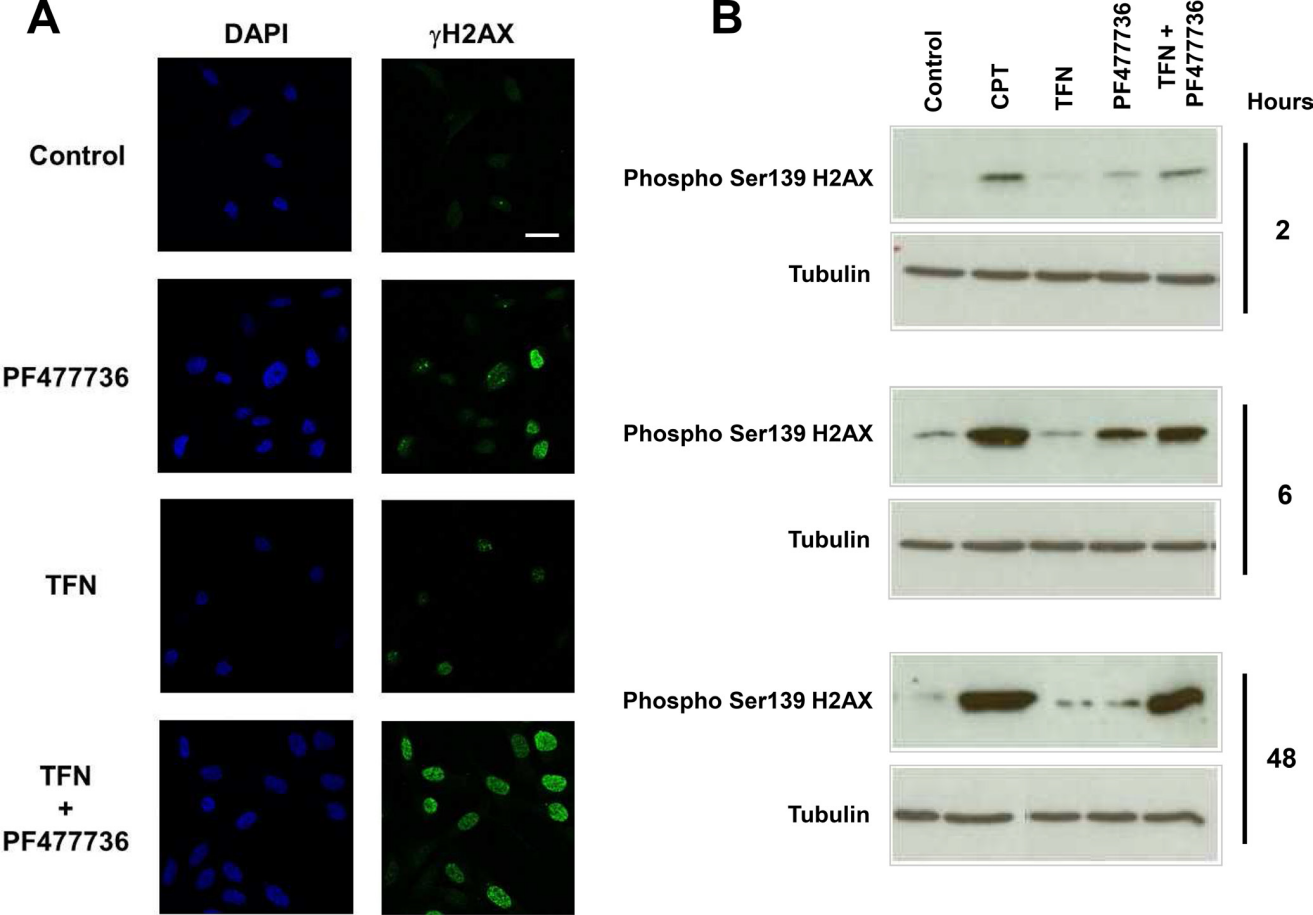
The combination of teriflunomide and PF477736 induces H2AX phosphorylation on serine 139 (γH2AX) in SUM159 triple negative breast cancer cell line (**A**) Immunofluorescence of DNA (blue) and γH2AX (green) in SUM159 cells that were exposed to either 25 μM TFN, IC10 PF477736 (2.5 μM) or the combination of these compounds. Scale bar, 20 μm (**B**) Western blotting analysis of H2AX phosphorylation on serine 139 (γH2AX) in cell lysates prepared 2, 6 and 48 hours after the beginning of the exposure to either compound, their combination or 0.1 μM positive control camptothecin (CPT).

**Figure 9:**
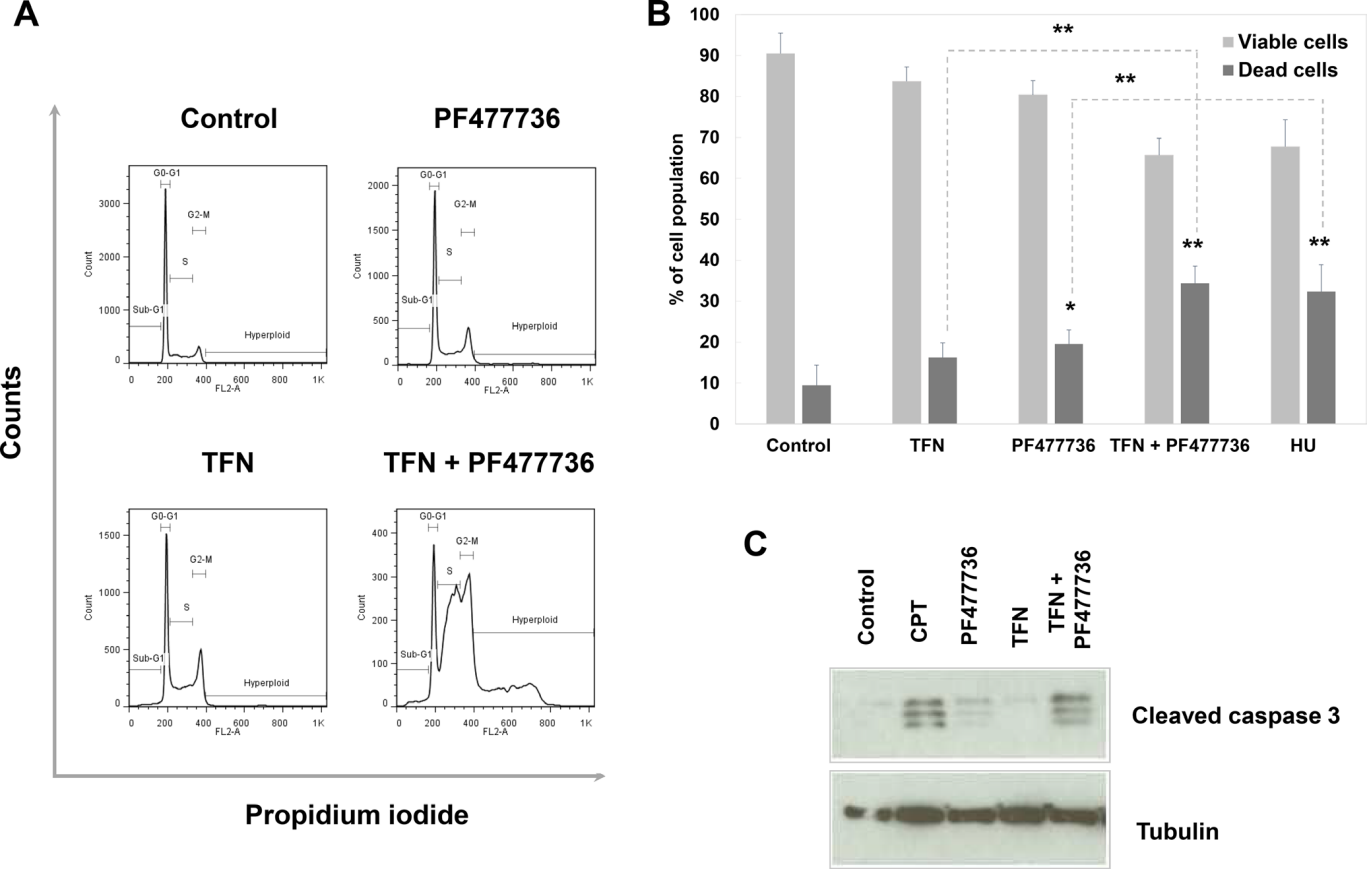
The combination of teriflunomide and PF477736 is cytotoxic in SUM159 triple negative breast cancer cell line (**A**) Representative flow cytometry analysis of cell cycle distribution in SUM159 cells collected 48 hours after the beginning of exposure to vehicle, 25 μM TFN, IC10 PF477736 (2.5 μM), or their combination at the same concentrations. (**B**) Flow cytometry analysis for apoptosis/ necrosis. Cells were exposed to vehicle, 5 mM hydroxyurea as a positive control, 25 μM TFN, IC10 PF477736 or their combination, collected and stained with annexin V/7-AAD. Quantitation was performed with FlowJo software. Results are expressed as mean values ± SD of three independent experiments. *p < 0.05 and **p < 0.01 as determined by two-tailed unpaired *t*-test. (**C**) Western blotting analysis of caspase 3 cleavage in cell lysates prepared 72 hours after the beginning of the exposure to either compound, their combination or 0.1 μM positive control camptothecin (CPT).

Strikingly, SUM159 cells were strongly γH2AX positive as early as 4 hours upon exposure to the combination as illustrated in Figure 8A. Furthermore western blotting analysis (Figure 8B) showed that this phosphorylation event occurred to an extent that was comparable to the one induced by exposure to DNA damaging agent camptothecin at 48 hours. This indicates that the association of DHODH and Chk1 inhibitors was highly effective in inducing DNA damage in these fast-proliferating TNBC cells. At 48 hours this TFN + IC10 PF477736 combination resulted in a significant cell accumulation in S and G2/M phases along with the appearance of a population of hyperploid cells (Figure 9A). As described with transformed mouse embryonic fibroblasts, this was also associated with a significant increase in the percentage of dead cells (Figure 9B) as compared with matched controls or each individual drug (*p* = 0.0046 *vs* DHODH inhibition; *p* = 0.0089 *vs* Chk1 inhibition). Western blotting experiments confirmed a significant induction of caspase 3-dependent apoptosis at 72 hours in cells exposed to the combination as compared with either inhibitor alone (Figure 9C). The induction of a substantial caspase 3-dependent cell death was also observed when Chk1-depleted cells were exposed to teriflunomide (Supplementary Figure 9).

Altogether these data confirmed that combining IC10 Chk1 inhibitor with a DHODH inhibitor can convert two cytostatic effects into cytotoxicity in both mouse and human cancer cells in culture.

### Pharmacological effect of the combination of DHODH and Chk1 inhibitors in SUM159 xenografts

In order to assess the *in vivo* relevance of this combination the anti-tumour activities of TFN and PF477736 were then tested as single therapies or in sequential combination (TFN then PF477736) in SUM159 xenografts in nude mice (Supplementary Figure 10A and 10B). Paclitaxel was used as a reference treatment. The tested PF477736 regimen was consistent with those used in previous studies and well tolerated [36, 39]. Furthermore the fractionated administration (twice a day injections) of that compound was reported as more effective than a single injection [36]. TFN dosing used in the present study was chosen in accordance with regulatory safety reports [40] showing that daily oral administration of 5 mg/kg TFN over a prolonged period of time should not induce repeated dose toxicity including drug-induced carcinogenesis. Unfortunately at that dose no significant effect was observed on tumour growth when teriflunomide was used either as a single therapy or in combination with PF477736 while as expected paclitaxel was effective (*p* = 0.02). Immunohistochemistry analyses (Supplementary Figure 10C) were consistent with this moderate effect and showed a slight but not significant reduction in tumour Ki67 staining on day 3 of the protocol upon treatment with the combination as compared with the control group and no significant change in cleaved caspase-3 levels was noticed (data not shown). This negative result suggested this safe dose of teriflunomide was suboptimal to produce an anti-tumour effect in that model. Of note we tested the chronic administration of higher doses that turned out to be highly toxic within days and therefore not suitable for *in vivo* administration.

## DISCUSSION

Conventional chemotherapies have been combined with Chk1 inhibitors in order to increase their cytotoxicity through the abrogation of S and G2/M Chk1-dependent DNA damage checkpoints. This was proposed as a synthetic lethal approach to kill p53 mutant tumour cells whilst sparing p53 wild type normal cells which still rely on their p53-dependent G1 checkpoint to cope with DNA damage [41]. Following this strategy the *in vitro* and *in vivo* combination of Chk1 inhibition with antimetabolites, such as nucleoside analogues gemcitabine or cladribine, was reported as more effective than with other classes of DNA damaging agents [42]. Here we demonstrate that a p53-independent similar effect can be achieved by combining a Chk1 inhibitor with an inhibitor of the one enzyme which controls the mitochondrial step of *de novo* pyrimidine synthesis.

Our growth assays in mouse embryonic fibroblasts first confirmed the selective antiproliferative effect of DHODH inhibition in transformed or immortalised cells with defective p53-dependent checkpoints. Strikingly exposure with as little as IC10 Chk1 inhibitor enhanced the antiproliferative effect induced by two unrelated DHODH inhibitors (teriflunomide or IPP-A017-A04). The choice of very low-effect (IC10) PF477736 concentration was motivated to minimise off-target effects reported with checkpoint kinase inhibitors [34]. Initially set up in p53-deficient transformed MEFs this strategy led to alterations in cell cycle distribution and viability. This was associated with the appearance of DNA damage, hyperploid DNA content, DNA damage and replication stress response with Chk1 phosphorylation on serine 345 [43, 44], followed with massive caspase-3 dependent cell death. Importantly each separate compound had only a moderate impact on cell cycle distribution without signs of loss of viability. In brief, this suggests the conversion of two cytostatic effects into cytotoxicity. This was exacerbated in SUM159 and HCC1937 triple negative breast cancer cell lines which were not at all sensitive to teriflunomide alone in our experimental setting. Interestingly, the DHODHi+Chk1i combination had a weak and non-significant effect on BT549 and HCC38 cell lines. This encourages us to explore the cellular and molecular characteristics that may account for the observed variability in the pharmacological effect between these cell models. This should allow identification and selection of potential responders to this combination therapy.

Altogether our observations are coherent with recent studies in which combining the nucleoside analogue gemcitabine and Chk1 inhibition resulted in prolonged interphase with increased DNA damage, unresolved replication stress, entry of cells with damaged DNA into mitosis and cell death [44, 45]. Beside the novel synthetic lethality revealed by this study, our work also further highlights the relevance of targeting DHODH in cancer. Recent studies reported DHODH inhibition as a promising pharmacological strategy in multiple myeloma, neuroblastoma, melanoma models and acute myeloid leukemia [20–22, 25]. Our present work adds triple negative breast cancer (TNBC) to this list and might pave the way for novel pharmacological strategies to treat this cancer which is currently resistant to most conventional therapeutic warfare. Interestingly, in p53-proficient cancer cells, it has been clearly shown that DHODH inhibition induced p53 activation and p53-dependent cell death [23, 24]. We are now showing that this strategy of targeting DHODH is also effective in a p53-deficient background provided that Chk1 function is inhibited. This observation fits with the fact that Chk1- and p53-dependent checkpoints are partially redundant and that activation of Chk1 -often through overexpression- is the way p53-deficient cancer cells are still able to elicit a partial DNA damage response [34]. Such Chk1 overexpression is frequently observed in TNBC as compared with other malignant subtypes, benign lesions and normal breast tissue [46] suggesting TNBC patients may be eligible for future therapeutic strategies aiming at inhibiting both DHODH and Chk1.

As promising as this new strategy may appear through our *in vitro* studies, its application in an *in vivo* context warrants further optimisation. The preclinical pilot experiment reported in this manuscript did not allow us to draw any conclusion but identified major technical caveats to alleviate before proceeding, starting with the narrow margin of safety of teriflunomide and its poorly documented tissue accessibility and clearance in mice. In brief, we first tested a dose of teriflunomide that was consistent with previous studies reporting tumour growth delay in carcinoids upon treatment with prodrug leflunomide [47] but animals experienced acute toxicity. This regimen had therefore to be drastically revised to remain within the ethical boundaries and a lower dose of teriflunomide was chosen in order to be consistent with data from the FDA safety report [40]. Overall toxicity was assessed throughout this new protocol and no animal had to be euthanised whatever the treatment in our study. At a lower dose of teriflunomide, which was not reported to induce repeated dose toxicity including drug-induced carcinogenesis (5 mg/kg/day), no significant effect was observed on tumour growth with or without PF477736. This limited pharmacodynamic effect suggests that either the amount of DNA damage generated in tumour cells did not reach a level sufficient to result in cytotoxicity due to underdosing (or a suboptimal administration schedule), or that inhibition of *de novo* pyrimidine synthesis was circumvented to some extent by the pyrimidine salvage pathway which contributes to resistance to chemotherapies such as antimetabolites [48]. Nevertheless the first hypothesis is the most plausible since highly proliferating cells need cooperation of *de novo* synthesis and salvage pathway to meet the increasing demand of pyrimidine nucleotides. Moreover the murine model might not be the most appropriate to evaluate the pre-clinical efficacy of a teriflunomide-based chronic treatment since TFN plasma half-life was reported to range between 18 and 37 hours in mouse, rat and dog [40] while it reached 15-18 days in MS patients [49]. Using a new generation of DHODH inhibitors endowed with improved pharmacokinetic parameters along with increased tumour accessibility and safety may circumvent this pitfall.

In summary, the present study showed the effective pharmacological targeting of DHODH and Chk1 in murine and human p53-deficient transformed cells through the generation of massive and irreparable DNA damage. Optimising the *in vivo* combination regimen within the ethical boundaries is necessary before this strategy can be considered as a suitable alternative to conventional chemotherapies.

## MATERIALS AND METHODS

### Chemicals

The active metabolite of leflunomide, teriflunomide (A771726, 2-cyano-3-hydroxy-N-(4-(trifluoromethyl)phenyl)but-2-enamide, see Supplementary Figure 1), was purchased from Selleck Chemicals LLC (Houston, TX). DHODH inhibitor IPP-A017-A04 (5-cyclopropyl-2-(4-(2,6-difluorophenoxy)-3-isopropoxy-5-methyl-1H-pyrazol-1-yl)-3-fluoropyridine, see Supplementary Figure 1) was synthesised according to the published procedure [10] and Chk1 inhibitor PF477736 ((2R)-2-Amino-2-cyclohexyl-N-[2-(1-methyl-1H-pyrazol-4-yl)-6-oxo-5,6-di hydro-1H-[1,2]diazepino[4,5,6-cd]indol-8-yl]-acetamide, see Supplementary Figure 1) was purchased from Axon Medchem (Groningen, Netherlands). Drugs were kept at −20°C as 10 mM stock solutions in DMSO. Paclitaxel was supplied by Fresenius-Kabi. Camptothecin, gemcitabine, hydroxyurea, sulforhodamine B, 7-AminoActinomycin D, propidium iodide and RNase A were purchased from Sigma Aldrich.

### Cell culture

Primary, p53^KO^ and p53^KO^ mouse embryonic fibroblasts transformed by HaRas^V12^ [35] were grown in DMEM-GlutaMax supplemented with 10% fetal bovine serum, 100 μg/ml streptomycin and 100 units/ml penicillin. SUM159 triple negative breast cancer cells were obtained from Asterand Bioscience, UK, and grown in Ham's F-12 medium supplemented with 5% fetal bovine serum, 10 μg/ml insulin, 1 μg/ml hydrocortisone, 100 μg/ml streptomycin and 100 units/ml penicillin. HCC1937 (ATCC-CRL-2336), HCC38 (ATCC-CRL-2314) and BT-549 (ATCC HTB-122) triple negative breast cancer cells were obtained from American Type Culture Collection. HCC1937 and HCC38 cells were grown in RPMI supplemented with 10% fetal bovine serum, 100 μg/ml streptomycin and 100 units/ml penicillin. BT-549 cells were grown in Dulbecco Modified Eagle Medium supplemented with 10% fetal bovine serum, 100 μg/ml streptomycin and 100 units/ml penicillin. All cell types were grown at 37°C in a humidified atmosphere containing 5% CO_2_ and were regularly checked for the absence of mycoplasma contamination.

### Growth inhibition assay

Inhibition of cell growth for each individual drug or combinations was assessed using the sulforhodamine B technique [50]. Two thousand SUM159 or BT-549 breast cancer cells, HaRas^V12^ transformed p53^KO^ or p53^KO^ mouse embryonic fibroblasts, fifteen hundred HCC38 cells, one thousand HCC1937 cells or four thousand primary mouse embryonic fibroblasts were seeded onto 96-well plates on day 1. On day 2, cells were exposed for 24 hours to increasing concentrations of teriflunomide, IPP-A017-A04 or PF477736. Medium was then replaced and cells grown for three doubling times, fixed with 12.5% trichloroacetic acid in PBS, washed with water and stained with 0.4% sulforhodamine B in 1% acetic acid. Each well was washed 5 times with acetic acid to remove unbound dye and protein-bound SRB was extracted with 10 mM Tris base pH 10.5. Optical density (540 nm) in each well was measured using a Tecan^®^ infinite 200 PRO multimode reader. Average +/− SD values from three independent experiments were plotted using GraphPad Prism software.

### Small Interfering RNA Transfection

Transformed mouse embryonic fibroblasts were transfected with 700 pmoles of either a mix of 4 target-specific 19-25 nt small interfering RNA (siRNA) for mouse Chk1 (Santa Cruz Biotechnology sc-29270), or SASI_Mm01_00087610 and SASI_Mm01_00087610 MISSION^®^ siRNA for mouse DHODH (Sigma Aldrich), or 1400 pmoles of the combination of Chk1 and DHODH siRNA. SUM159 cells were transfected with 700 pmoles of either SASI_Hs02_00326304 and SASI_Hs02_00326304_AS MISSION^®^ siRNA for Human Kinase Chk1 (Sigma Aldrich), or SASI_Hs01_00246561 and SASI_Hs01_00246561_AS MISSION^®^ siRNA for human DHODH (Sigma Aldrich) and 1400 pmoles of the combination of Chk1 and DHODH siRNA. A 20-25 nt siRNA (Santa Cruz Biotechnology sc-37007) was used as a control. Transfection of siRNA duplexes was performed for 5 hours using lipofectamine RNAiMAX transfection reagent (Invitrogen) according to the manufacturer's instructions. Samples were collected 48 hours post-transfection and probed for Chk1 and DHODH protein levels.

### Flow cytometric analysis of cell cycle phase distribution

Exponentially growing cells were exposed to teriflunomide, IPP-A017-A04, PF4777736, 0.1 μM camptothecin or 5 mM hydroxyurea (used as positive controls) or the combination of a DHODH inhibitor and PF477736 for 24 hours then grown in drug-free medium. At each time point (8, 24 and 48 hours after the beginning of the exposure), cells were washed once with ice-cold PBS, trypsinised and counted. One million cells per sample were fixed in ice-cold 70% ethanol and stored at -20°C until analysis. They were then washed in PBS before being suspended in 0.5 ml staining solution (5 μg/ml 7-AminoActinomycin D or 25 μg/ml propidium iodide, 200 μg/ml ribonuclease A in PBS) and incubated at 37°C for 30 min. Analysis of 10000 events was performed on a FACSCalibur flow cytometer (Becton Dickinson). DNA fluorescence was collected in linear mode using a doublet discrimination gate and cell cycle distribution was analysed using FlowJo software (Tree Star). Each experiment was performed three times.

### Quantification of cell death using multiparametric flow cytometry

Exponentially growing cells were exposed to teriflunomide, IPP-A017-A04, PF4777736, 0.1 μM camptothecin, 5 mM hydroxyurea or the combination of a DHODH inhibitor and PF477736 for 24 hours then grown in drug-free medium. At each time point, cells were washed with PBS, trypsinised, pooled with floating cells and counted. The annexin V and 7-AAD (or propidium iodide) dual labelling of apoptotic cells was conducted using the annexin V-FLUOS Staining Kit from Roche Applied Science according to the manufacturer's instructions. Two million cells were washed twice with PBS and once with binding buffer (10 mM HEPES, 140 mM NaCl, 5 mM CaCl2, pH 7.4). They were then incubated at room temperature in the dark for 30 min in 2 μl annexin-V-Fluos reagent and 5 μg/ml 7-AAD (or 25 μg/ml propidium iodide) in binding buffer. Analysis of 20000 events was performed on a FACSCalibur flow cytometer. DNA fluorescence was collected in logarithmic mode and cell viability was quantitated using FlowJo software. The population of dead cells was calculated as the sum of events in upper (left + right) and lower right quadrants in “annexin-V-FITC” *vs* “7-AAD” dot plots. Each experiment was performed three times.

### Immunoblotting

Exponentially growing cells were exposed to teriflunomide, IPP-A017-A04, PF4777736, 0.1 μM camptothecin, 40 μM gemcitabine, or the combination of a DHODH inhibitor and PF477736 for up to 24 hours. At each mentioned time point, cells were collected, washed with ice-cold PBS and pellets were resuspended in lysis buffer (50 mM Tris HCl pH 7.4 containing 100 mM NaCl, 50 mM NaF, 40 mM β-glycerophosphate, 5 mM EDTA, 1% Triton X-100, 1 mM sodium orthovanadate, 100 μM PMSF, 1 μM leupeptin, 1 μM pepstatin A and 1 μM aprotinin) and incubated on ice for 20 minutes. Lysates were then centrifuged for 10 min at 13000 rpm and 4°C and supernatant protein concentrations were determined using the bicinchoninic acid assay.

Twenty-five microgram proteins were resolved in 7.5% polyacrylamide gels and transferred onto nitrocellulose membranes (Whatman). Membranes were incubated overnight with either mouse monoclonal anti-Chk1 (G4, Santa Cruz Biotechnology), rabbit anti-Phospho-Chk1 (Ser345 or Ser296, Cell Signalling Technology), mouse monoclonal anti-Chk2 (clone 7, Upstate), mouse monoclonal anti-DHODH (E-8, Santa Cruz Biotechnology) or mouse monoclonal anti-β-actin (clone AC-15, Sigma) antibodies. For cleaved caspase-3 and gamma-H2AX assays, 15 microgram proteins were resolved in 15% polyacrylamide gels. Membranes were incubated overnight with either anti-phospho-histone (Ser139) H2AX (1/500, Merck Millipore), cleaved caspase-3 (Asp175) (Cell Signalling Technology) or anti-tubulin (Sigma) rabbit antibodies. Signals were visualised using horseradish-conjugated antibodies and Luminata Chemiluminescent detection substrate (Millipore).

### Immunofluorescence

Exponentially growing cells were seeded in chamber slides, exposed to teriflunomide, IPP-A017-A04, PF4777736, 0.1 μM camptothecin or the combination of a DHODH inhibitor and PF477736 for up to 24 hours. Cells were washed twice with PBS, incubated in 4% paraformaldehyde in PBS for 10 minutes, washed in PBS for 5 minutes, blocked with PBS containing 2% bovine serum albumin and 0.5% triton X-100, and incubated overnight at 4°C with anti-phospho-histone (Ser139) H2AX (1/500, Merck Millipore) antibody. Slides were washed, incubated with a FITC AlexaFluor 488-conjugated goat anti-rabbit IgG antibody for 1 hour at room temperature, and washed in PBS. Slides were mounted and image acquisition was performed on a LSM780 confocal microscope (Zeiss, Germany) at the Montpellier RIO Imaging facility (Campus CNRS route de Mende). Images were processed using ImageJ software.

### *In vivo* study and drug administration

All experimental procedures were conducted in compliance with recommendations issued by the *Comite d’Ethique Regional Languedoc-Roussillon* - *Comite d’Ethique pour l’Experimentation Animale* and the Guidelines for the welfare of animals in experimental neoplasia. Female Swiss *nu/nu* mice (Charles River Laboratories) were housed under clean room conditions in sterile individually ventilated cages. Animals received sterile irradiated chow and water *ad libitum*.

Ten million SUM159 cells were inoculated subcutaneously in 150 μl of 50:50 medium/matrigel (BD biosciences) in the right hind flank region of 6-week old mice. When tumours reached approximately 100 mm^3^ animals were randomly assigned into five groups of 10 mice. Mice were then administered with (a) vehicle or (b) oral teriflunomide at a dose of 5 mg/kg once a day for 28 days, (c) 7.5 mg/kg PF477736 twice daily (6-hour interval) by *i.p* injections on days 2, 9, 16 and 23, (d) teriflunomide and PF477736 according to the same schedules as single regimens and (e) 20 mg/kg paclitaxel by *i.p* injection on days 1, 8 and 15. Animals were weighed twice a week and tumour volumes were calculated by caliper measurements once every three days using the following formula: V = (length × width × height × π/6). Animals were euthanised when tumour burden reached 1500 mm^3^.

### Immunohistochemistry analysis

Five tumour-bearing mice were monitored in each group on days 2 and 3 of the protocol for pharmacodynamic assessment of the aforementioned regimens by immunohistochemistry. Tumours were harvested 24 or 48 hours after the beginning of each treatment and were fixed with formalin, all sections were counterstained with hematoxylin and eosin. Samples were also probed for Ki67 and cleaved caspase-3 levels. Quantitative analysis of Ki67 section staining was performed using ImageJ software and caspase-3 cleavage was quantitated using Aperio ImageScope software.

### Statistical analyses

*In vitro* data expressed as mean values +/- SD of three independent experiments were analysed using Statview 5.0 software (Informer Technologies) using a two-tailed unpaired Student *t* test. *In vivo* data were analysed according to a non-linear mixed-effect model using Stata ver.13 (Stata Corporation, College Station, TX, USA). Kaplan-Meier survival curves were compared using the Log-rank test.

## SUPPLEMENTARY MATERIALS FIGURES AND TABLES


