# Supplementary material for: Checkpoint kinase 1 inhibition sensitises transformed cells to dihydroorotate dehydrogenase inhibition
Source: Oncotarget. 2017 Jul 12;8(56):95206–22. doi: 10.18632/oncotarget.19199 (PMC5707016; doi:10.18632/oncotarget.19199)
Supplement: Supplementary file 1 [file oncotarget-08-95206-s001.pdf]

Checkpoint kinase 1 inhibition sensitises transformed cells to dihydroorotate dehydrogenase inhibition

SUPPLEMENTARY MATERIALS

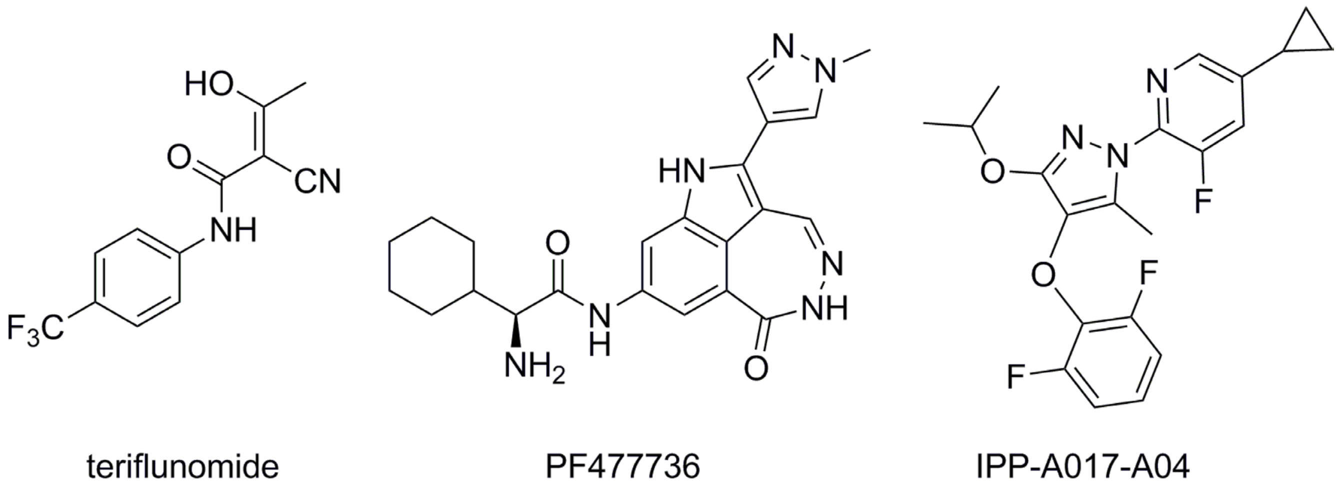

Supplementary Figure 1: Structures of teriflunomide (TFN), PF477736 and the new DHODH inhibitor 5-cyclopropyl-2-(4-(2,6-difluorophenoxy)-3-isopropoxy-5-methyl-1H-pyrazol-1-yl)-3-fluoropyridine (IPPA017A04).

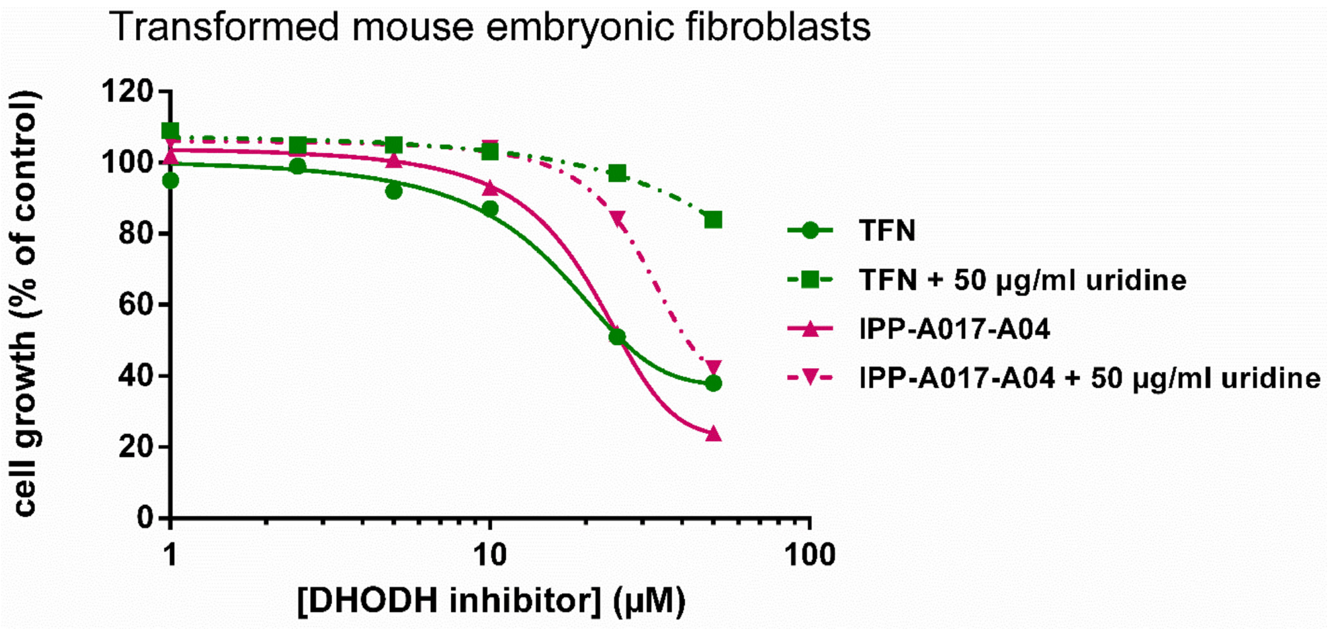

Supplementary Figure 2: Rescue of DHODH inhibitor effect by precursors of pyrimidine biosynthesis. Transformed mouse embryonic fibroblasts were exposed for 24 hours to increasing concentrations of teriflunomide (TFN) or IPP-A017-A04 and grown in drugfree medium for three doubling times. Whenever indicated, uridine (50 µg/ml) was maintained within the medium throughout the cell growth assay.

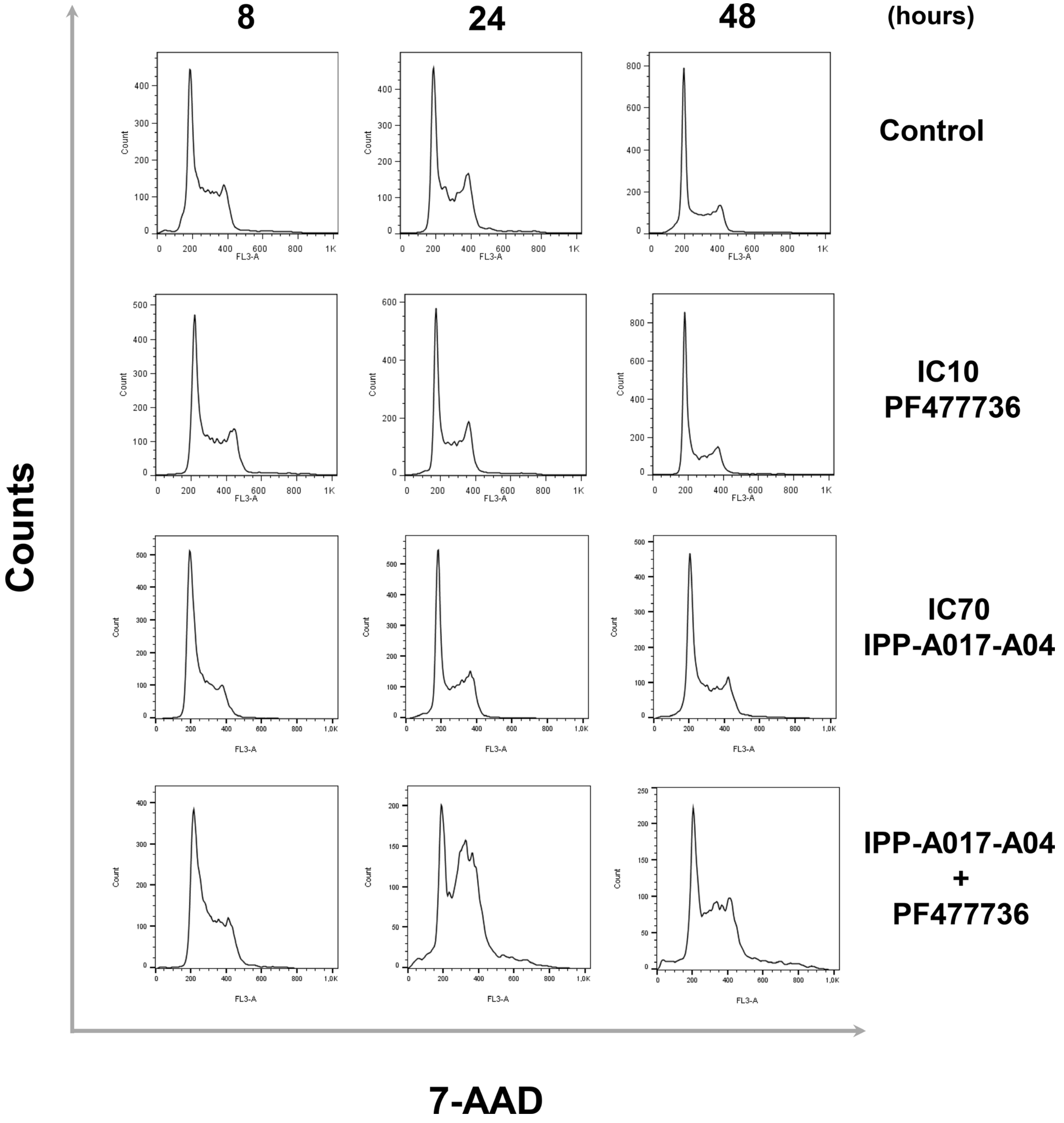

Supplementary Figure 3: The combination of IPP-A017-A04 and PF477736 results in significant cell cycle perturbations in transformed mouse embryonic fibroblasts. Flow cytometry analysis for cell cycle distribution in transformed mouse embryonic fibroblasts up to 48 hours after the beginning of the exposure. Cells were exposed to vehicle, IC70 IPPA017A04 (22 µM), IC10 PF477736 (0.7 µM) or their combination for 24 hours, collected and stained with 7-AAD. Results are representative of three independent experiments.

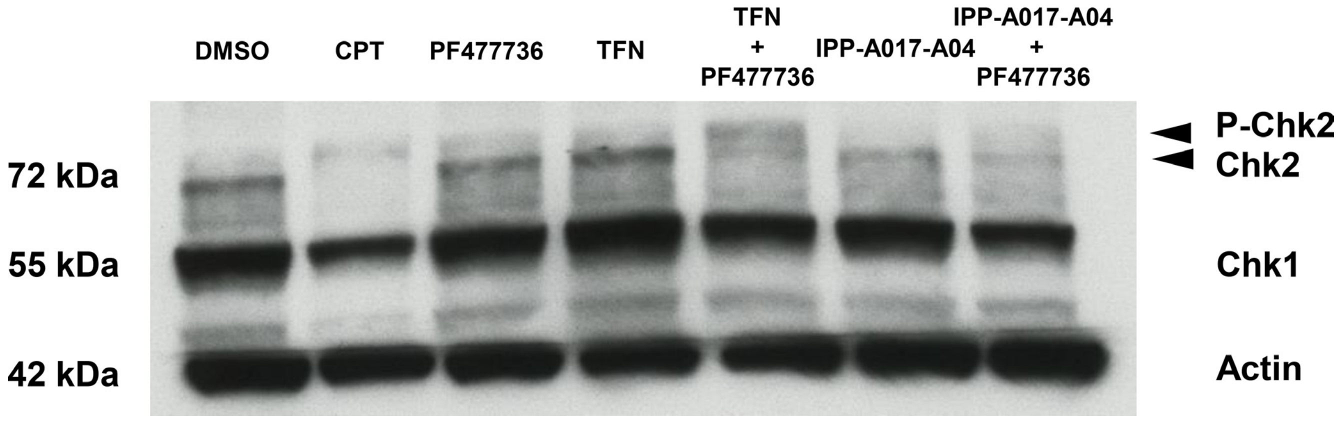

Supplementary Figure 4: The combination of teriflunomide and PF477736 triggers Chk2 phosphorylation in transformed mouse embryonic fibroblasts. Western blotting analysis of total Chk2 and total Chk1 levels in cell lysates prepared 8 hours after the beginning of the exposure. Cells were exposed to vehicle, 0.1 µM camptothecin (CPT), IC10 PF477736 (0.7 µM), IC70 TFN (10 µM) ± IC10 PF477736 and IC70 IPPA017A04 (22 µM) ± IC10 PF477736. Membrane was cut above the 45 kDa marker then upper and lower parts were probed for Chk1 + Chk2 total levels and beta-actin respectively.

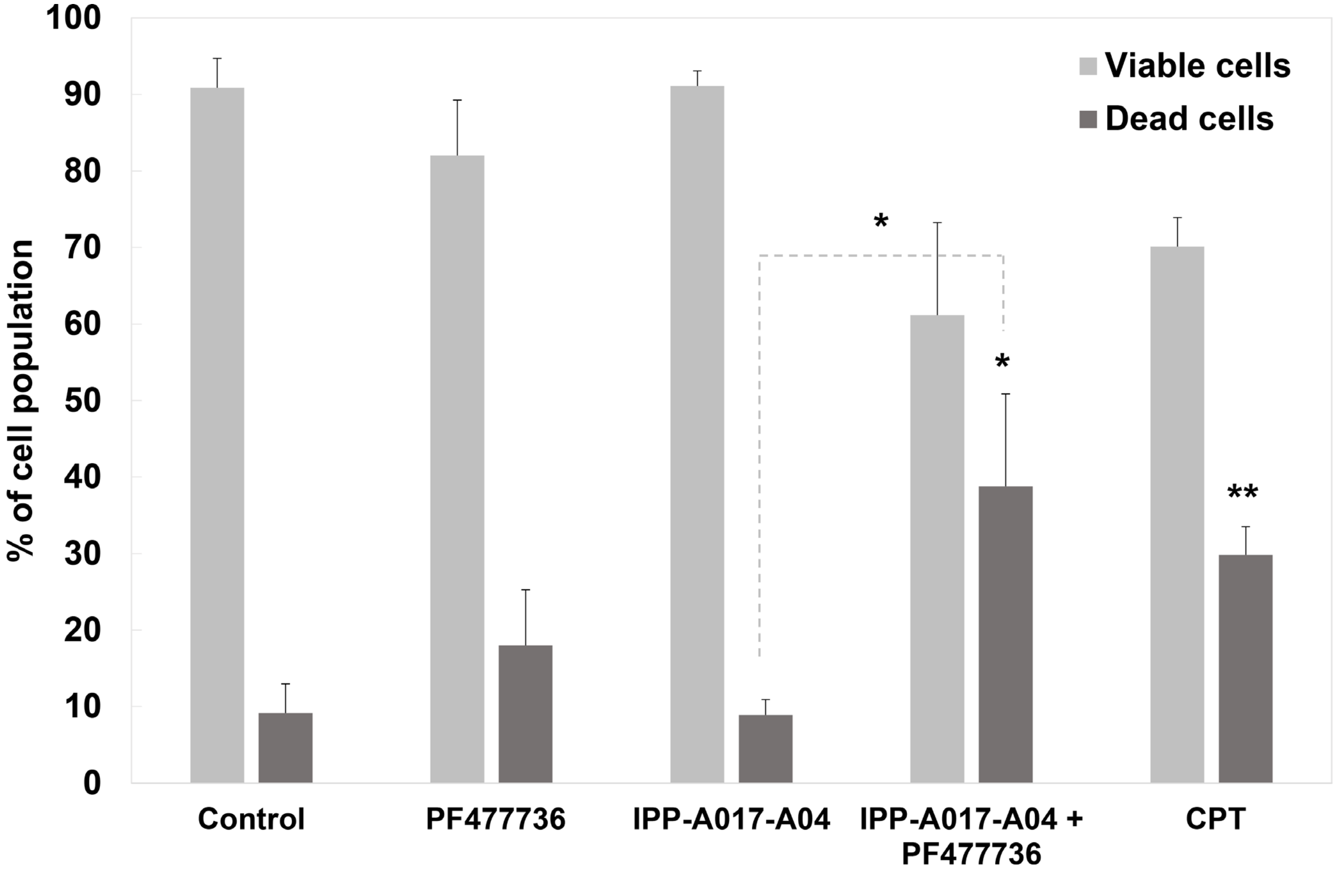

Supplementary Figure 5: The combination of IPP-A017-A04 and PF477736 is cytotoxic in transformed mouse embryonic fibroblasts. Flow cytometry analysis for apoptosis/necrosis. Cells were exposed to vehicle, 0.1 µM camptothecin (CPT), IC70 IPPA017A04, IC10 PF477736 or their combination for 24 hours, collected 48 hours after the beginning of the exposure and stained with annexin V-FITC /7-AAD. Quantitation was performed with FlowJo software. Results are expressed as mean values ± SD of three independent experiments. *$p < 0.05$, **$p < 0.01$ as determined by two-tailed unpaired t-test.

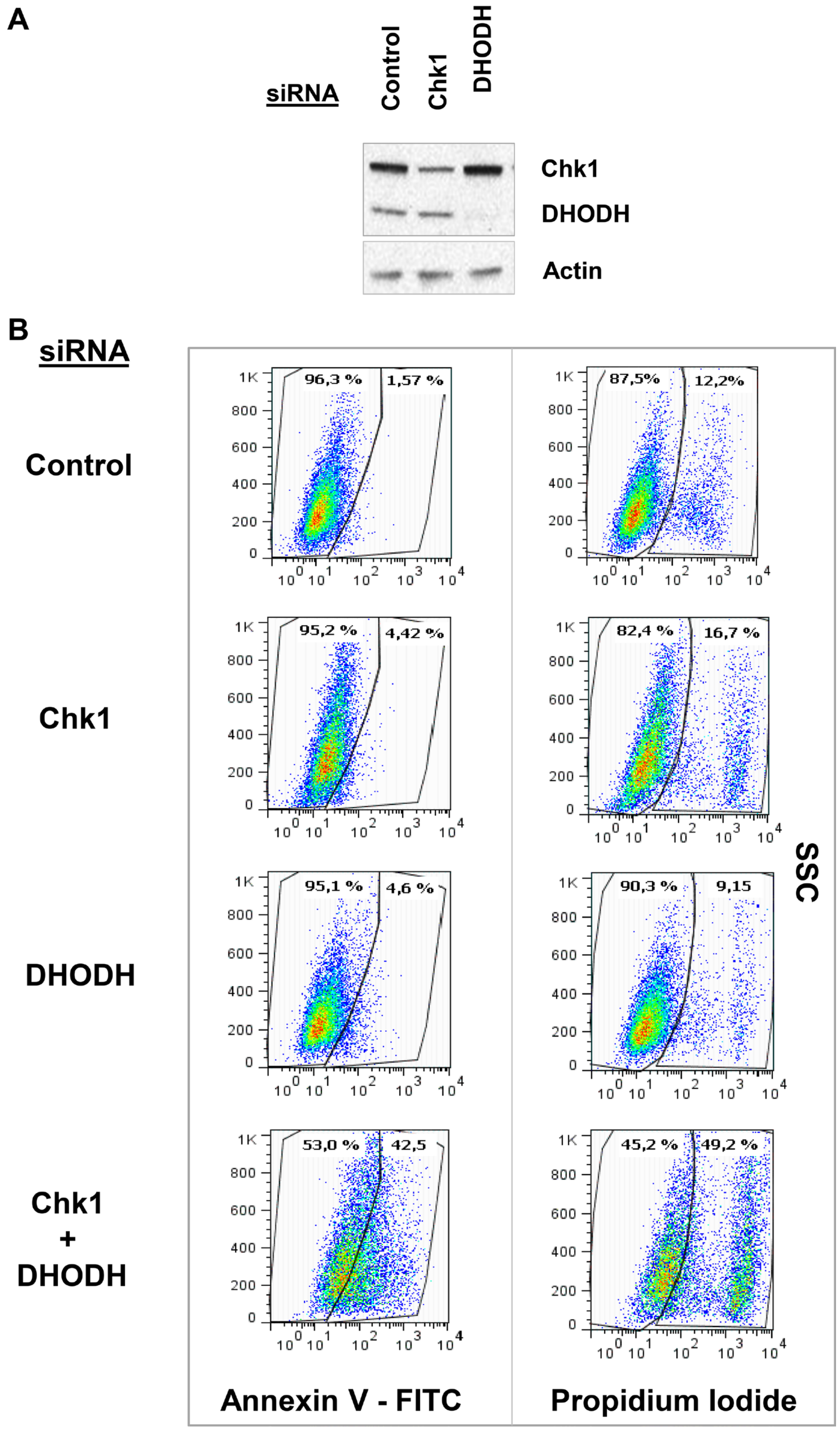

Supplementary Figure 6: Dual DHODH and Chk1 depletion results in cell death in transformed mouse embryonic fibroblasts. (**A**) Cells were transfected with the indicated siRNAs for 5 hours and collected 48 hours after the onset of depletion. DHODH and Chk1 protein levels were detected by western blotting and β-actin was used as loading control. (**B**) Flow cytometry analysis for apoptosis / necrosis in cells that were collected 48 hours posttransfection and stained with annexin V-FITC/propidium iodide. Quantitation was performed with FlowJo software.

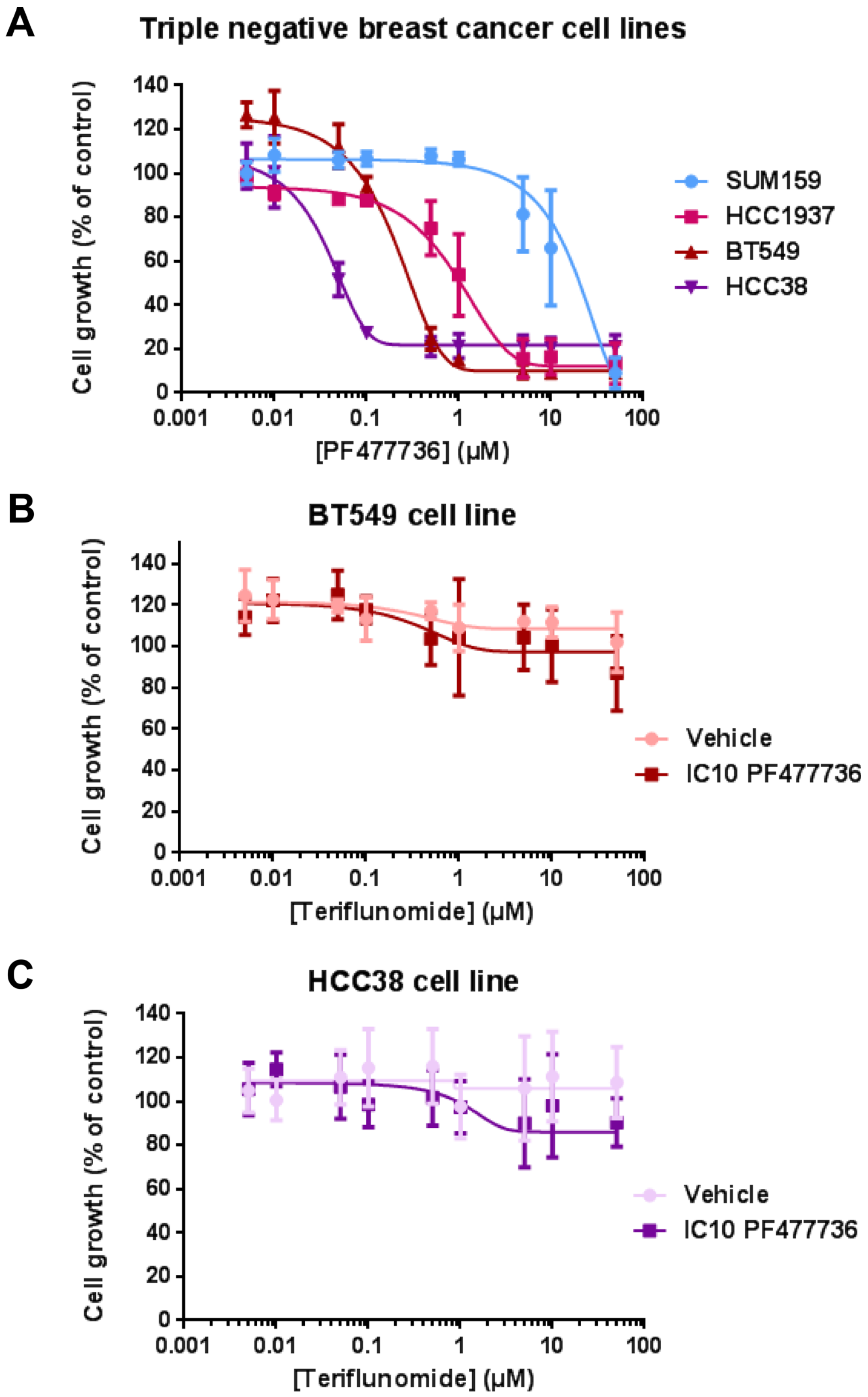

Supplementary Figure 7: Pharmacological activity of PF477736 as a single agent or in combination with teriflunomide in triple negative breast cancer cell lines. (**A**) SUM159, HCC1937, BT549 and HCC38 cells were exposed to increasing concentrations of PF477736 for 24 hours and grown in drug-free medium for three doubling times. Mean ± SD, *n* = 3 independent experiments. (**B**) BT549 and (**C**) HCC38 cells were exposed for 24 hours to increasing concentrations of teriflunomide ± IC10 PF477736 (0.05 µM and 0.02 µM respectively, added 30 minutes after the beginning of exposure to TFN) and grown in drug-free medium for three doubling times. Mean ± SD, *n* = 3 independent experiments.

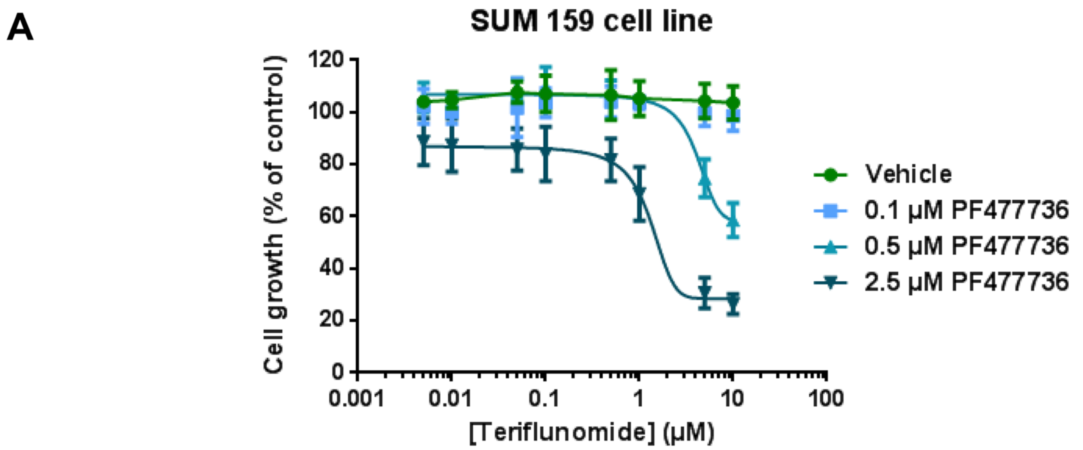

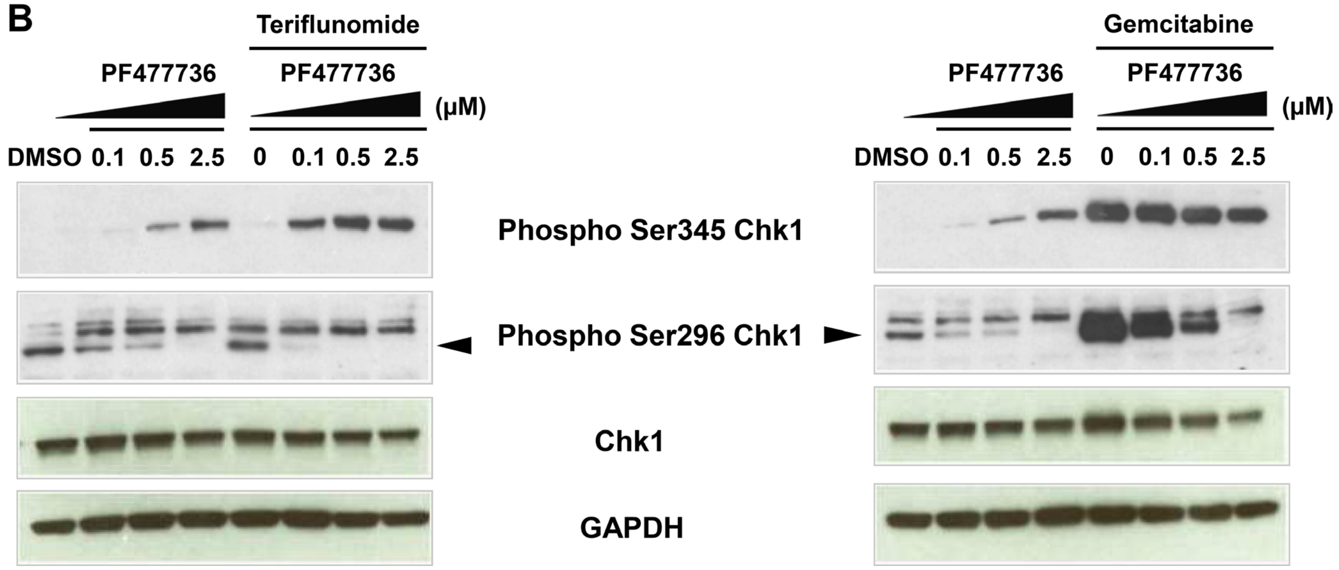

Supplementary Figure 8: The combination of teriflunomide and PF477736 results in an increased and concentration-dependent antiproliferative effect in SUM159 triple negative breast cancer cell line. (**A**) SUM159 cells were exposed for 24 hours to increasing concentrations of teriflunomide followed with either 0.1 µM, 0.5 µM or 2.5 µM PF477736 (or vehicle solution) which were added 30 minutes after the beginning of the exposure to DHODH inhibitor; cells were then grown in drug-free medium for three doubling times. Results are expressed as mean values ± SD of three independent experiments. (**B**) Western blotting analysis of total Chk1 levels and Chk1 phosphorylation on serines 296 and 345 in SUM159 cell lysates which were prepared 4 hours after the beginning of the exposure. Left panel: cells were exposed to either vehicle, 0.1 µM, 0.5 µM or 2.5 µM PF477736 alone or in combination with 25 µM teriflunomide. Right panel: cells were exposed to either vehicle, 0.1 µM, 0.5 µM or 2.5 µM PF477736 alone or in combination with 40 µM gemcitabine as a positive control for DNA damage induction.

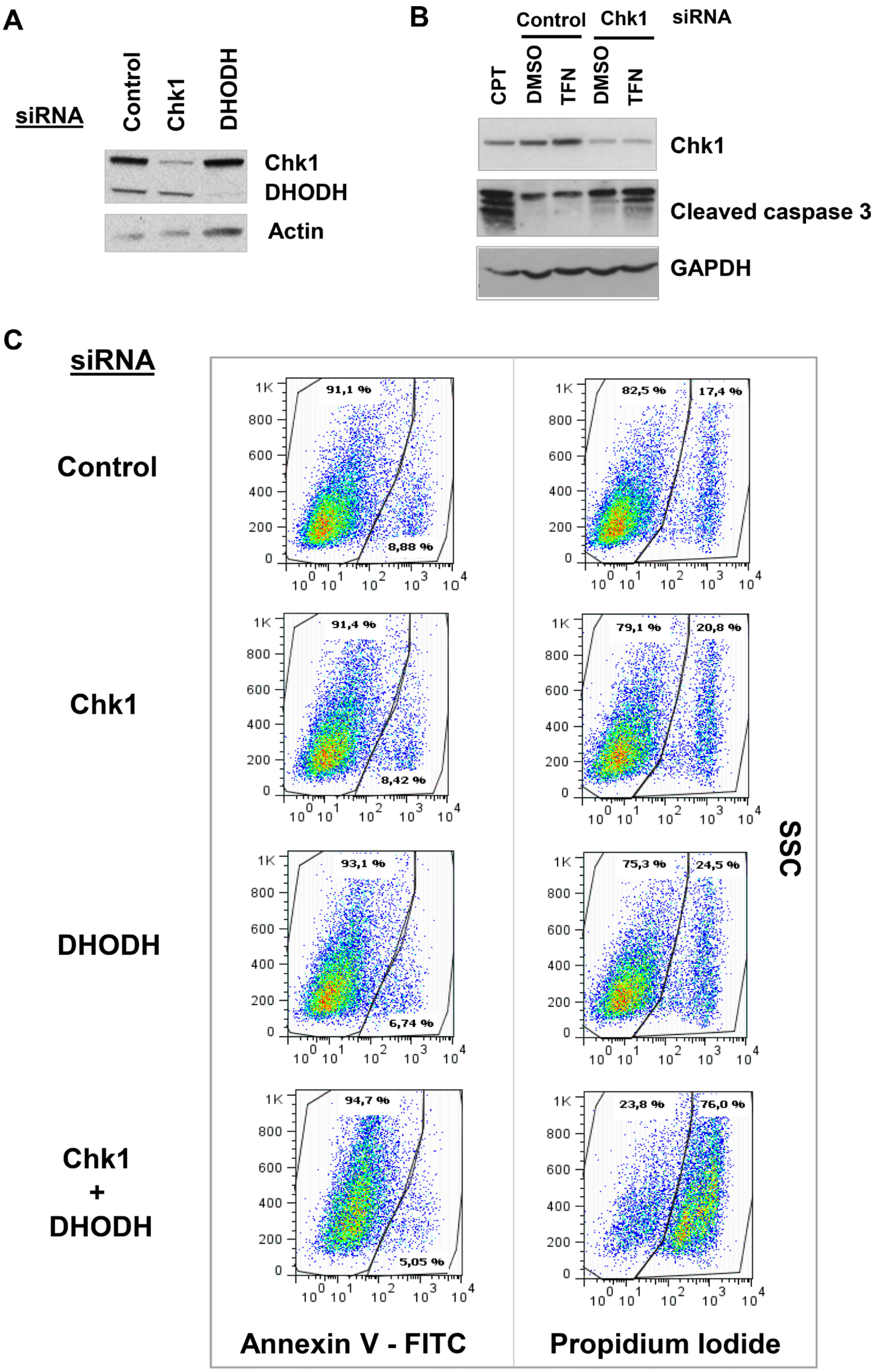

Supplementary Figure 9: Dual DHODH and Chk1 loss of function results in cell death in transformed mouse embryonic fibroblasts. (**A**) SUM159 cells were transfected with the indicated siRNAs for 5 hours and collected 48 hours after the onset of depletion. DHODH and Chk1 protein levels were detected by western blotting and β-actin was used as loading control. (**B**) Flow cytometry analysis for cell death in cells that were collected 48 hours post transfection and stained with annexin V-FITC / propidium iodide. Quantitation was performed with FlowJo software. (**C**) Exposure to teriflunomide results in cell death in Chk1-depleted SUM159 cell line. Cells were transfected with Chk1 siRNA then exposed to 25 μM TFN for 24 hours and collection was performed 48 hours after the onset of Chk1 depletion. Cleaved caspase-3 and Chk1 protein levels were detected by western blotting and GAPDH was used as loading control.

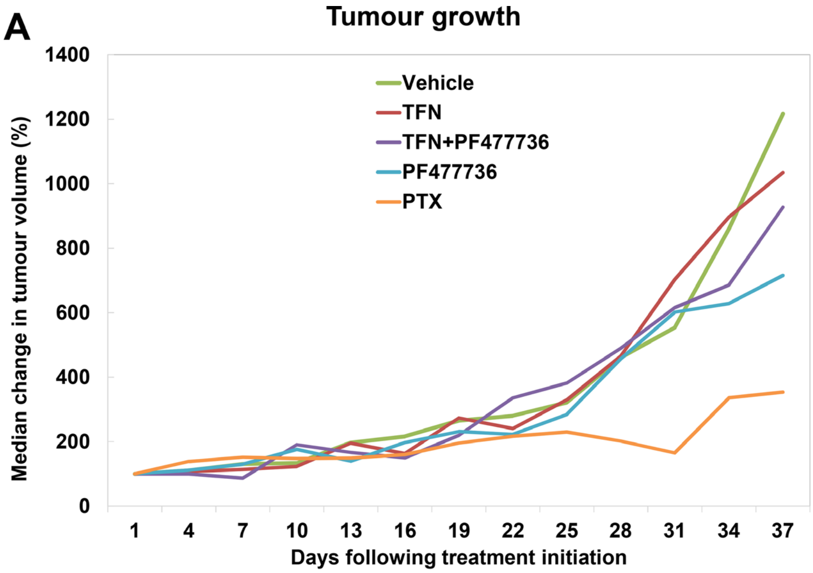

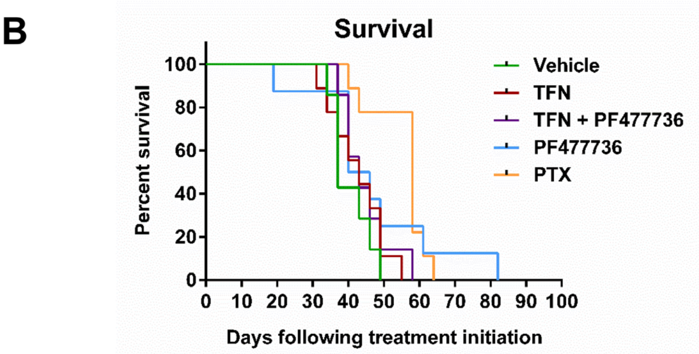

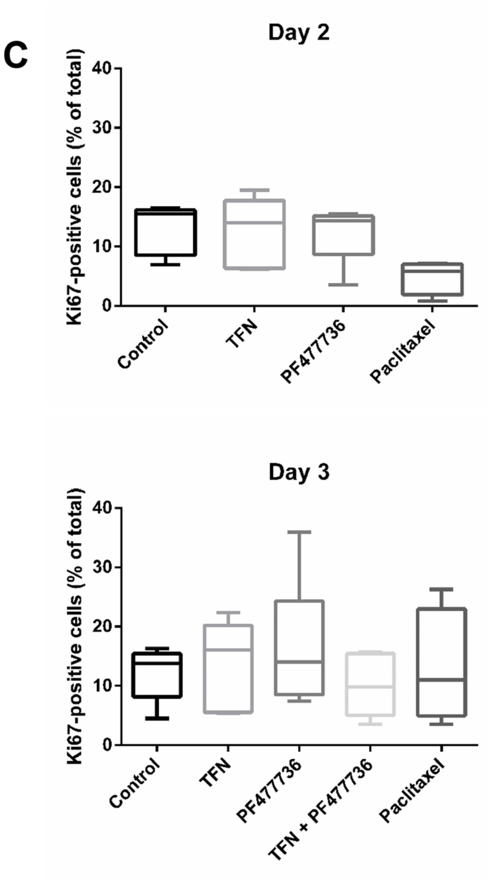

Group	Vehicle	TFN	TFN + PF477736	PF477736	PTX
Median survival (days)	37	43	43	43	58

Supplementary Figure 10: Effect of teriflunomide and PF477736 in combination in SUM159 xenografts. (**A**) Median relative tumour growth from treatment initiation up to the day the first animal reached the predefined endpoint. Swiss *nu/nu* mice were administered with (green) vehicle or (red) oral teriflunomide (TFN) at a dose of 5 mg/kg once a day for 28 days, (blue) 7.5 mg/kg PF477736 twice daily (6-hour interval) by *i.p* injections on days 2, 9, 16 and 23, (purple) TFN and PF477736 according to the same schedules as single regimens and (orange) 20 mg/kg paclitaxel by *i.p* injection on days 1, 8 and 15. Mice were weighed twice a week and tumour volumes were calculated by caliper measurements once every three days using the following formula: V = (length × width × height × π/6). *N* = 8, 10, 7, 8 and 9 animals for control, TFN, TFN + PF477736, PF477736 and paclitaxel groups respectively after censoring. (**B**) Overall survival represented as Kaplan-Meier curve. Mice were euthanised when tumour burden reached 1500 mm^3. (**C**) Semi-quantitative analysis of IHC Ki67 staining on days 2 and 3 of the protocol in groups of 5 animals.
